# Continent-island boundary and environment-shaped evolution in the marine amphipod *Ampithoe marcuzzii* complex (Crustacea: Eumalacostraca: Ampithoidae)

**DOI:** 10.1038/s41598-023-51049-5

**Published:** 2024-01-05

**Authors:** Tammy Iwasa-Arai, Silvana G. L. Siqueira, Thadeu Sobral-Souza, Fosca P. P. Leite, Sónia C. S. Andrade

**Affiliations:** 1 Departamento de Biologia Animal, Instituto de Biologia, Universidade Estadual de Campinas, Campinas, Brazil; 2 Departamento de Genética e Biologia Evolutiva, Instituto de Biociências, Universidade de São Paulo, São Paulo, Brazil; 3 Departamento de Botânica e Ecologia, Universidade Federal do Mato Grosso, Cuiabá, Brazil

**Keywords:** Molecular ecology, Biodiversity

## Abstract

Marine amphipods are crustaceans that lack a larval phase and consequently have low dispersion rates. Despite that, these crustaceans present a remarkable ability to be transported by rafting on natural floating substrata, especially macroalgae, where they find shelter, food and a mating ground. The species *Ampithoe marcuzzii* is widely distributed throughout the western Atlantic Ocean. Here, it was used as a model to study seascape genomics and phylogeography in invertebrates with low dispersion capacities. We anticipated that the lineages would present isolation-by-distance patterns. However, surface currents and other abiotic variables could facilitate connectivity among distant sites. Based on mitochondrial and nuclear genes, SNPs, and environmental associations, we observed the presence of a species complex within *A. marcuzzii,* separating mainland and insular populations. Each species showed an independent evolutionary history, with a strong latitudinal population structure and evidence of isolation-by-distance and isolation-by-environment, characterizing the 'continent' species. Historical expansion and environmental variables were observed associated with the southeastern population, and ecological niche modeling corroborated the region as a paleorefuge. Conversely, populations from 'islands' presented complicated evolutionary histories, with closer localities genetically isolated and distant localities connected. These findings indicate that insular populations with low dispersion capacity might be more susceptible to spatial connectivity by floating substrata and to changes in surface currents. In contrast, mainland populations might be more vulnerable to local climate changes due to lack of gene flow.

## Introduction

Macroalgal beds, one of the primary environments formed in coastal areas, are characterized by their high productivity and structural complexities that provide food resources and reproductive sites for a vast diversity of species and shelter from predators and adverse abiotic factors^1^. Among the most representative species that make up the macroalgal beds are those of the genus *Sargassum* C. Agardh, which have a wide distribution in the Atlantic Ocean and form dense and extensive meadows, representing about 80% of the cover and biomass of algae in the infralittoral rocky shores^2^. The Sargasso Sea, a characteristic conglomerate of macrophytes in the Northwest Atlantic Ocean, is mainly formed by pelagic floating masses of *Sargassum natans* (L.) Gaillon and *S. fluitans* (Børgesen) Børgesen^3^ that harbor a unique ecosystem^4^. Recently, changes driven by upwelling off West Africa during the Northern Hemisphere winter and by the Amazon River discharge are intensifying recurrent blooms in the tropical Atlantic and Caribbean Sea, denominated the 'great Atlantic *Sargassum* belt' (GASB^5^). These events could be causing the arrival of exotic species and compromising the ecosystem balance^4^.

The fauna associated with macrophyte beds is mainly composed of crustaceans of the superorder Peracarida Calman, 1904, which are characterized by having a marsupium formed by oostegites^6^. The order Amphipoda Latreille, 1816 corresponds to one of the most diverse within Peracarida and constitutes the most representative group of fauna associated with macrophyte banks^7^. Due to their direct development, such crustaceans are expected to have a low dispersal capacity, thus assuming a high level of endemism^8^. However, according to^9^, peracarid crustaceans are highly suitable for colonization by rafting on floating organisms since they can persist on the drifting substrates and retain their offspring on the parental float.

The amphipod fauna associated with the species of *Sargassum* is represented by over 30 species^7^. However, several species of amphipods common in this association have aroused discussions among taxonomists due to notable intrapopulation morphological variations^10^. Thus, it is possible that there is an underestimation of the richness of the amphipod fauna, based on phenotypic plasticity, and each of these species' names may represent species complexes^10^. The herbivorous species *Ampithoe marcuzzii* Ruffo, 1954, originally described from Los Roques, Venezuela, is relatively abundant in *Sargassum* banks off the Florida^11^, Brazil^12^ and Atlantic oceanic islands^13^; however, its evolutionary history remains uncertain.

The distinctive evolution on oceanic islands is recognized by the high endemicity caused by isolation^14,15^, and the diversity of isolated places, particularly marine peracarid species, is mainly shaped by dispersion^13^. In this sense, the marine communities inhabiting coastal areas and islands may differ in response to local biotic and abiotic factors^16^. The emergence of oceanic islands from the sea bottom, without any land connection, might have allowed a few specimens to arrive, therefore, colonization depends upon current systems and specific dispersal capabilities, as the definition of “Darwinian islands”^17,18^. In the case of peracarid crustaceans with low specific dispersal capacities with a primary wide distribution^16,19,20^, rafting might have contributed to population connectivity up to > 5000 km, substantially affecting evolutionary processes^21^.

Patterns previously observed for peracarid crustaceans in the Macaronesian and Webbnesian oceanic islands show that, despite the proximity with the continent, islands may harbour unique lineages^16^. Consequently, high endemic peracarid diversification was found in these archipelagosin a community level, with clear geographic concordance among close islands^16^.

In this study, we expect that isolation-by-distance (IBD) might play a role in the genetic structuring of *Ampithoe* due to the its low dispersion capability, while a disruption in the geographic structuring could be evidence of rafting on the coastline. Therefore, we aim to present the evolutionary history of the amphipod *Ampithoe marcuzzii* as a model to study seascape genomics and phylogeography in invertebrates with low dispersion capacities in the western Atlantic Ocean, coupling genetic and environmental data to understand the current and historical gene flow, to then broadly discuss the evolution and biogeography of marine invertebrates with low dispersion capacities along the coast and on the dynamics of isolated island connectivity.

## Results

Over 5000 km of *Ampithoe marcuzzii’*s *sensu latu* (*s.l.*) distribution were sampled, totaling 21 localities along the Brazilian mainland, continental islands, oceanic islands and Caribbean (Table 1).

**Table 1:** Sampling data from *Ampithoe marcuzzii s.l*.

Acronym	Locality	Domain	Latitude	Longitude	Date	Depth (m)	Macroalgae	# specimens sequenced
Continent								
FLE	Flecheiras Beach, Trairi	Continent—NE	−3.21691	−39.26537	12/11/21	1	*Sargassum* sp.	4
PCR	Pedra Rachada Beach, Paracuru	Continent—NE	−3.39661	−39.01099	10/11/21	1	*Sargassum* sp.	7
CAU	Pacheco Beach, Caucaia	Continent—NE	−3.68584	−38.63456	14/11/21	1	*Hypnea* sp.	8
NAT	Pirambúzios Beach, Natal	Continent—NE	−6.006086	−35.10539	17/11/18	5	*Dictyota* sp.	12
CSA	Calhetas Beach, Cabo de Santo Agostinho	Continent—NE	−8.34378	−34.94334	15/11/18	2	*Sargassum* sp. and *Dictyota* sp.	10
FOR	Forte Beach, Mata de São João	Continent—NE	−12.58016	−38.00077	30/12/18	1	*Sargassum* sp. and *Dictyota* sp.	10
BOI	Moreré Beach, Boipeba Island	Continent—E	−13.62898	−38.88702	07/01/19	1	*Sargassum* sp. and *Dictyota* sp.	12
TRA	Coqueiros Beach, Trancoso	Continent—E	−16.59698	−39.08787	26/12/18	1	*Sargassum* sp. and *Dictyota* sp.	4
ABR	Redonda Island, Abrolhos Archipelago	Continent—E	−17.96835	−38.70926	12/01/19	3	*Sargassum* sp. and *Dictyota* sp*.*	1
CF	Peró Beach, Cabo Frio	Continent—SE	−22.86632	−41.97831	25/01/21	1	*Sargassum* sp.	15
SPD	Domingas Dias Beach, Ubatuba	Continent—SE	−23.49694	−45.14718	2016	1	*Sargassum* sp.	10
SINO	Pedra do Sino Beach, Ilhabela	Continent—SE	−23.75153	−45.34941	2016	1	*Sargassum* sp.	10
Islands								
SM	Baie Rouge, Saint Martin	Saint Martin	18.071326	−63.121448	19/07/19	1	*Sargassum* sp.	3
CDM	Secret Beach, Portsmouth, Dominica	Islands—LA	15.546467	−61.473314	08/07/19	1	*Sargassum* sp.	3
CDM	Soufrière, Dominica	Islands—LA	15.228475	−61.36082	17/07/19	1	*Sargassum* sp.	10
SSV	San Sauveur, Dominica	Islands—LA	15.403329	−61.250248	12/07/19	1	*Sargassum* sp.	9
FN	Conceição Beach, Fernando de Noronha	Islands—OI	−3.839021	−32.415273	24/11/18	1	*Sargassum* sp.	10
FN	Porto Beach, Fernando de Noronha	Islands—OI	−3.836277	−32.404728	21/11/18	1	*Sargassum* sp.	9
FN	Sueste Beach, Fernando de Noronha	Islands—OI	−3.868175	−32.421794	20/11/18	1	*Sargassum* sp. and *Dictyota* sp.	2
RA	Falsa Barreta Pool, Rocas Atoll	Islands—OI	−3.856736	−33.801844	10/12/19	1	*Sargassum* sp. and *Dictyota* sp.	11
TR	Parcel Pool, Trindade Island	Islands—OI	−20.50502	−29.34430	05/02/19	5	*Dictyota* sp.	11

### SNPs prospection and data analysis

The sequenced libraries ranged from 312,772 to 12,261,962 reads per sample. After filtering and clustering steps, the average consensus read number was 26,284 (Supplementary Table S1). One 'merged' dataset was used for *Ampithoe marcuzzii s.l.,* in which 5,857 SNPs were recovered from 134 specimens, with 31.19% of total missing data (Supplementary Fig. S1). Two posterior datasets were built, with Saint Martin (SM) specimen removal and separation of the 'continent' and 'islands' lineages (defined in Table 1) for comparative population analyses, resulting in 21,126 SNPs, 72 specimens and 27.55% of total missing data, and 17,237 SNPs, 55 specimens and 16.09% of total missing data, respectively (Supplementary Figs. S2 and S3).

### Phylogenetic analyses and divergence time estimates

#### mtDNA and 28SRNA

**Figure 1:**
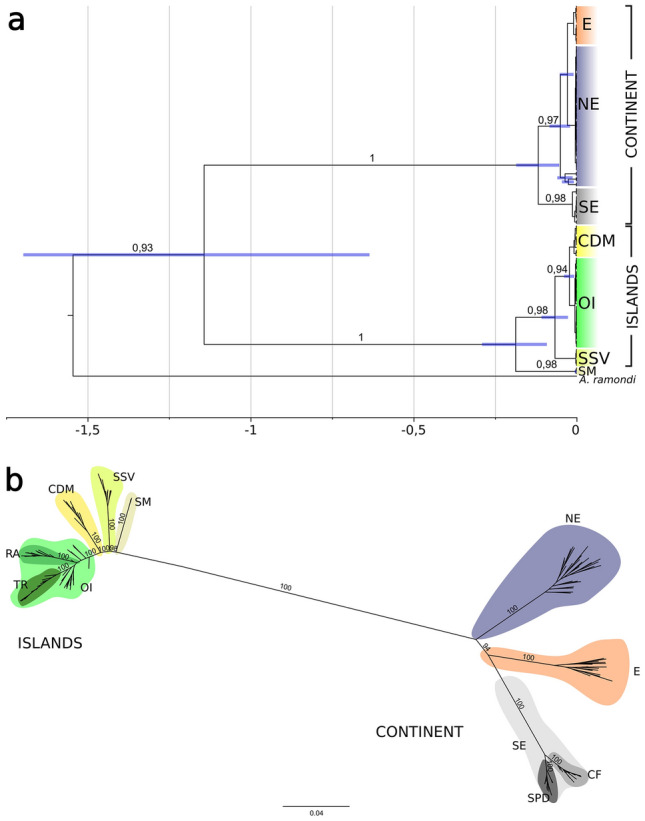
Phylogenetic trees of *Ampithoe marcuzzii s.l.* (**a**) Bayesian calibrated tree of *Ampithoe marcuzzii s.l.* using concatenated COI + 16SrRNA + 28SrRNA. Numbers above branches represent posterior probability values over 0.9. Node bars represent the 95% HPD interval. Time divergence bar is in a Mya scale. (**b**) Maximum likelihood tree recovered from 5857 SNPs. Bootstraps above 80% are shown above branches. *E* eastern Brazil, *NE* northestern Brazil, *SE* southeastern Brazil, *CDM* Caribbean Dominica, *OI* Brazilian oceanic islands (Rocas Atoll, Fernando de Noronha and Trindade), *SSV* San Sauveur (Dominica), *SM* Saint Martin.

Three distinct molecular operational taxonomic units (MOTUs) were identified by the phylogenetic tree and species delimitation analysis (Fig. 1a and Supplementary Fig. S5), showing a clear distinction between *A. marcuzzii s.l.* from the 'continent', 'islands', and SM*.* A 'continent' clade was recovered, with northeastern Brazil (NE) being a sister clade of eastern Brazil (E) and southeastern Brazil (SE) being the sister clade of E + NE. As shown in Fig. 1a, the multilocus tree of mtDNA + 28SrRNA was used to infer the divergence times within *Ampithoe marcuzzii s.l.* Specimens from the NE localities of Caucaia (CAU), Flecheiras (FLE) and Paracuru (PCR) clustered within NE, while Trancoso (TRA), in the eastern mainland, belonged to E. 'Islands' clade did not show islands’ structure patterns, clustering all the oceanic islands (OI) together, while separated from the Dominican clades of Caribbean Dominica (CDM) and San Sauveur (SSV) (Fig. 1).

According to our divergence time estimations, the 'continent' and 'islands' clades have modern divergence in the Pleistocene, around 1.14 Mya (95% HPD: 0.64–1.70, Fig. 1a, Supplementary Fig. S4). Within the 'continent', SE split from E + NE around 0.12 Mya (95% HPD: 0.05–0.19), while NE split from E in the Holocene, around 0.03 Mya (95% HPD: 0.02–0.08). The SM Caribbean population split from the 'islands' clade in the Pleistocene, around 0.19 Mya (95% HPD: 0.10–0.29), followed by the split of SSV around 0.07 Mya (95% HPD: 0.03–0.11), and the divergence of the oceanic islands (OI) from CDM in the Holocene, around 0.02 Mya (95% HPD: 0.01–0.02, Fig. 1a, Supplementary Fig. S4).

#### SNPs

Unrooted phylogenomic reconstruction agreed with the spatial distribution of *A. marcuzzii s.l.*, with the separation of the main clades into SE and NE continent, and one clade grouping the Brazilian OIs and the Dominican sites in the Lesser Antilles (LA, Fig. 1b). Moreover, phylogenomic analyses also revealed locality monophyly for several sampling sites, including one clade from the estuarine island of Boipeba (BOI), Praia do Forte (FOR) in the NE continent, Cabo Frio (CF) and Ubatuba (SPD) in the SE continent, the OIs of Trindade (TR) and Rocas Atoll (RA), CDM and SSV (Fig. 1b).

A highly supported clade named 'islands' was recovered, comprising a clade from the tropical oceanic island TR and another from the equatorial island RA. On the other hand, Fernando de Noronha (FN) was recovered as paraphyletic and including TR and RA clades (Fig. 1b), clustered together the three Brazilian OIs. The OIs are a sister clade of CDM. SSV is the only locality sampled in Atlantic Dominica and is the sister clade of OI + CDM, while SM is the sister clade of all remaining 'islands' specimens.

### Genetic diversity and population structure

Because of the low sampling of SM specimens, further analyses were performed for 'continent' and 'islands' only. Genetic diversity indexes slightly differed between the 'continent' and 'islands', with overall haplotype diversity (H) similar for the 'continent' and 'islands', and nucleotide diversity (θ_π_) between individuals being lower for 'continent' for COI (θ_π_ = 1.50) and SNPs (θ_π_ = 118.60), and higher for 16SrRNA (θ_π_ = 12.33) and 28SrRNA (θ_π_ = 0.12) (Supplementary Table S2). Overall θ_π_ values for the 'islands' varied from 0.00 for 28SrRNA to 260.24 for SNPs. Within the 'continent', greater diversity varied among markers, with values of 8.00 in Flecheiras (FLE) (NE) for COI, 57.60 in CF (SE) for 16SrRNA, 0.536 in Cabo de Santo Agostinho (CSA, NE) for 28SrRNA and 194.00 and 187.48 for SNPs in FOR (NE) and Abrolhos (ABR, E), respectively. Regarding the 'islands' clade, the highest values of θ_π_ also varied among localities, with values of 14.55 in CDM for COI, 31.72 in FN for 16SrRNA and 409.64 in RA for SNPs, while θ_π_ values were null for 28SrRNA (Supplementary Table S2). *F*_IS_ values across the loci were only significant for two localities in the 'island' dataset (RA = 0.093 and SSV = 0.382, p < 0.05, Supplementary Table S2).

Using the genetic clusters E, NE and SE for 'continent', and OI, CDM and SSV for 'islands' to perform a hierarchical AMOVA, the 'continent' results of all markers suggested that most of the total variance is retained among groups, with high significant Φ_ST_ and Φ_CT_ indices for all genes and the SNPs (Table 2). The 'islands' results recovered most of the total variance among groups for COI and 28SrRNA, while 16SrRNA and SNPs showed most of the variance within populations (Table 2).

**Table 2:** Hierarchical analysis of molecular variance (AMOVA) results from all genetic markers. Localities grouped according to lineages (Continent: NE, E and SE; Islands: CDM, SSV and OI).

	Among groups					Among populations within groups					Within populations				
	%	d.f	Variance components	ΦCT	*p*	%	d.f	Variance components	ΦSC	*p*	%	d.f	Variance components	ΦST	*p*
Continent															
COI	54.52	2	3.62	0.55	0.01	35.29	8	2.35	0.78	0.00	10.19	66	0.68	0.90	0.00
16SrRNA	52.04	2	1.51	0.52	0.01	7.23	4	0.21	0.15	0.01	40.73	50	1.19	0.59	0.00
28SrRNA	99.07	2	9.85	0.99	0.00	0.20	5	0.02	0.21	0.03	0.74	46	0.07	0.99	0.00
SNPs	68.20	2	28.86	0.68	0.00	5.88	6	25.92	0.19	0.00	2.49	135	10.97	0.74	0.00
Islands															
COI	65.33	2	4.41	0.65	0.10	3.64	2	0.25	0.11	0.00	31.03	46	2.09	0.70	0.00
16SrRNA	12.74	2	0.17	0.13	0.21	8.31	2	0.11	0.09	0.00	78.95	43	1.08	0.21	0.00
28SrRNA	97.83	2	13.89	0.98	0.11	2.17	2	0.31	1.00	0.00	0.00	31	0.00	1.00	0.00
SNPs	43.90	2	29.27	0.44	0.09	7.63	2	5.09	0.14	0.00	48.47	105	32.31	0.52	0.00

The pairwise *F*_ST_ suggests augmented differentiation between localities from different regions (i.e., NE, E and SE) in the 'continent' clade for all markers (Supplementary Fig. S5). Moreover, within the 'islands' clade, the highest values of *F*_ST_ were observed between SSV and TR for all markers (16SrRNA *F*_ST_ = 0.68; 28SrRNA *F*_ST_ = 1.00; COI *F*_ST_ = 0.69; Supplementary Fig. S4, Supplementary Table S3). The most conserved marker used, the nuclear 28SrRNA, presented higher pairwise *F*_ST_ values (28SrRNA *F*_ST_ = 1.00). In contrast, most variable SNPs recovered less differentiation between populations. Mantel tests with the SNP dataset for IBD were significant for the 'continent' (Mantel r^2^ = 0.71, *p* = 0.002), whereas no evidence of IBD was observed for the 'islands' (Mantel r^2^ = 0.06, *p* = 0.334).

#### mtDNA and 28SrRNA

**Figure 2:**
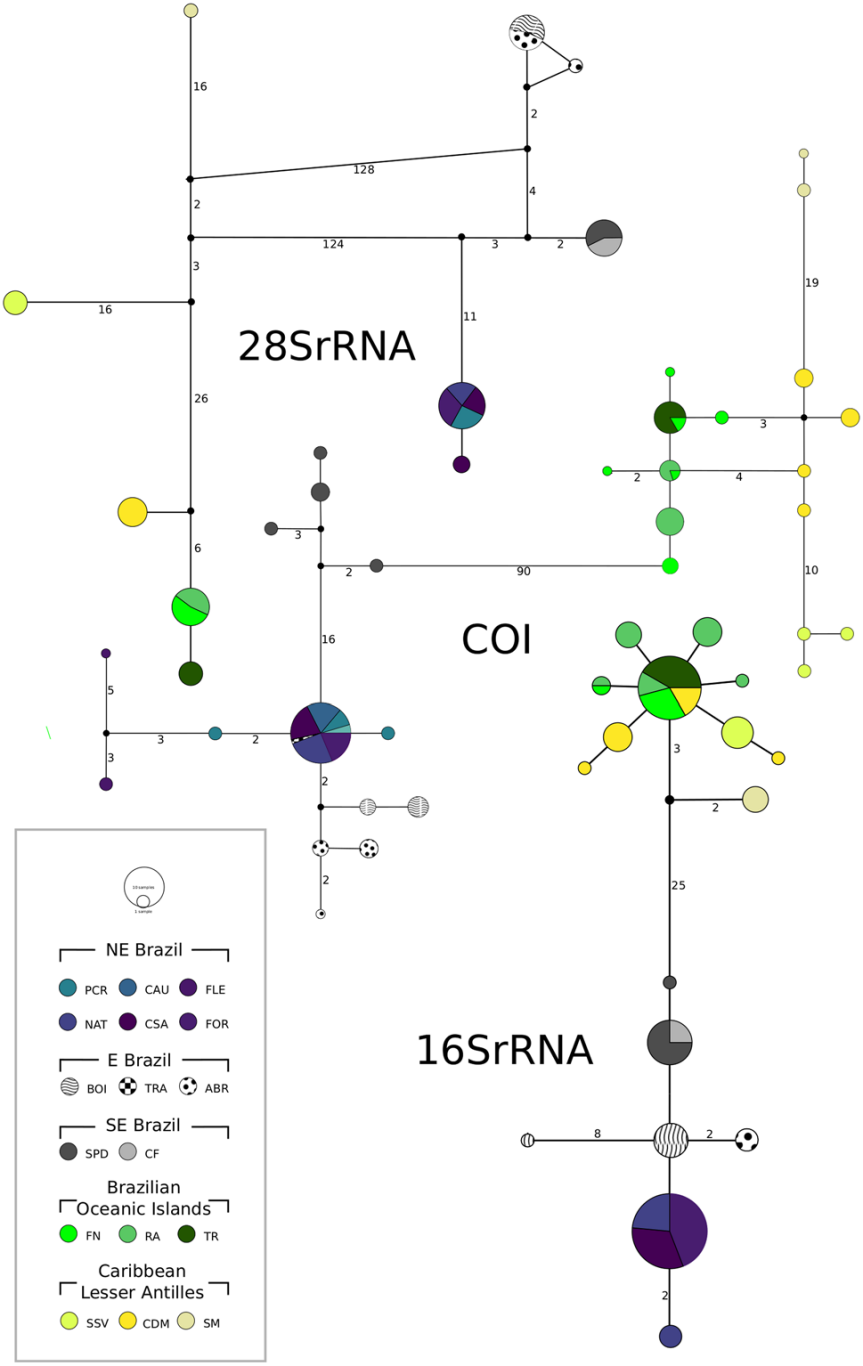
Minimum spanning network of 28SrRNA above, COI in the middle, and 16SrRNA below. Each node represents a haplotype, its size corresponds to the haplotype frequency and its colours indicate the locality. Black nodes represent inferred ancestral nodes. Numbers on branches represent mutational steps.

The haplotype networks of COI, 16SrRNA and 28SrRNA presented a clear geographic pattern with two sets of haplotypes separated by over 100 mutational steps in the 28SrRNA network (Fig. 2). These two main sets of haplotypes are consistent with the 'continent' and 'islands' clusters. In all networks, specimens from NE and SE localities are generally characterized by the presence of private haplotypes, with one shared haplotype between localities of the NE region in all network (Fig. 2). The population structure from region E in the continent diverged among networks, with unique haplotypes from ABR and BOI for COI and 16SrRNA and shared haplotypes between ABR and BOI for 28SrRNA (Fig. 2). The 'islands' clade, on the other hand, presented different network shapes, mainly constituted of private COI and 28SrRNA haplotypes, and a primary shared 16SrRNA haplotype (Fig. 2). Concerning COI, specimens from OI localities shared haplotypes and specimens from the LA presented private haplotypes. In turn, 16SrRNA had a primary haplotype shared by seven localities, including the OI and LA sites (Fig. 2).

#### SNPs

**Figure 3:**
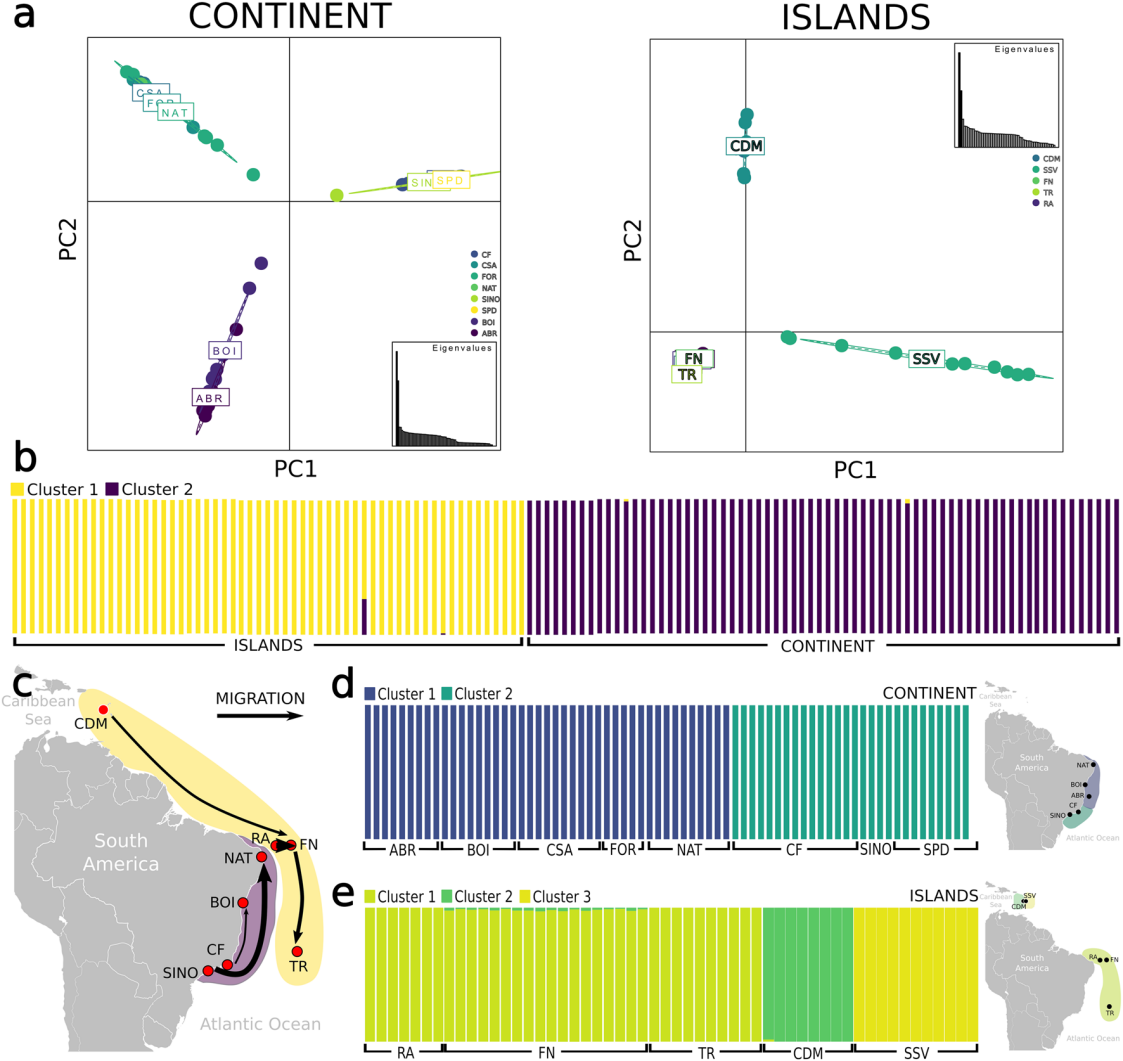
(**a**) PCA plots for ‘continent’ and ‘islands’ Ampithoe populations showing the first two axis for SNPs dataset. Dots represent individuals, with colours denoting the localities. (**b**) Structure results from *A. marcuzzii s.l.* dataset (k = 2). Each vertical bar corresponds to one individual, and the colour indicates the proportion of membership in each genetic cluster. (**c**) Main migration routes inferred from Fastsimcoal for SNPs. Arrows width correspond to comparative amount of migrants (see figures S15 and S16 for more information on migration rate). (**d,e**) Structure results from ‘continent’ (k = 2) and ‘islands’ (k = 3). Small maps show ‘continent’ and ‘islands’ geographic structure. *ABR* Abrolhos Archipelago, *BOI* Boipeba Island, *CF* Cabo Frio, *FOR* Forte Beach, *NAT* Natal, *SINO* Ilhabela, *SPD* Ubatuba, *FN* Fernando de Noronha, *RA* Rocas Atoll, *TR* Trindade Island, *CDM* Caribbean Dominica, *SSV* San Sauveur.

PCA and DAPC scatter plots of the SNPs showed a symmetric spatial distribution of clusters NE, E and SE within 'continent' for the first two PC axes (Fig. 3a and Supplementary Fig. S6), with the main separation of NE and E from SE in the first axis, and the separation of E from NE and SE on the second axis (Fig. 3a and Supplementary Fig. S6). Within the 'islands', the PCA's first axis separated the three genetic clusters of the OIs, CDM and SSV. In comparison, the second axis mostly separated CDM from the two remaining genetic clusters (Fig. 3a). Lastly, the DAPC analysis suggested four clusters, including two from SSV (Supplementary Fig. S7). The genotypic structure attribution recovered with SNPs resulted in two genetic clusters for the entire *Ampithoe marcuzzii s.l.* dataset, in strong agreement with the two proposed clades of 'continent' and 'islands' (Fig. 3b and Supplementary Fig. S8), except for one single individual (SSVS5F8) from the Atlantic Dominican locality of SSV that presented 28% of 'continent' cluster identity (Fig. 3b). A second Structure analysis with separated datasets into 'continent' and 'islands' recovered two and three genetic clusters, respectively (Fig. 3d, e). The 'continent' analyses revealed two Ks with the highest ΔK values: Structure analyses with K = 2 suggested complete separation of populations from NE + E and SE (Fig. 3d), whereas K = 3 separated E from NE (Supplementary Figs. S9 and S10). The 'islands' analyses were also geographically congruent, separating individuals into three genetic clusters corresponding to OIs, CDM and SSV samples (Fig. 3e and Supplementary Fig. S11).

### Demographic history and divergence times

#### mtDNA and 28SrRNA

The investigation of mtDNA demographic history was obtained from Tajima's D and Fu's Fs analyses. The results did not detect significant evidence of population expansion for most lineages, except for the OIs (D =  −2.69, *p* < 0.005, Supplementary Table S4). The estimates of variation in population size over evolutionary time based on the Bayesian skyline plots suggested that lineages' population sizes have remained constant for long period before declining over their recent evolutionary past (Supplementary Figs. S12–S15).

#### SNPs

**Figure 4:**
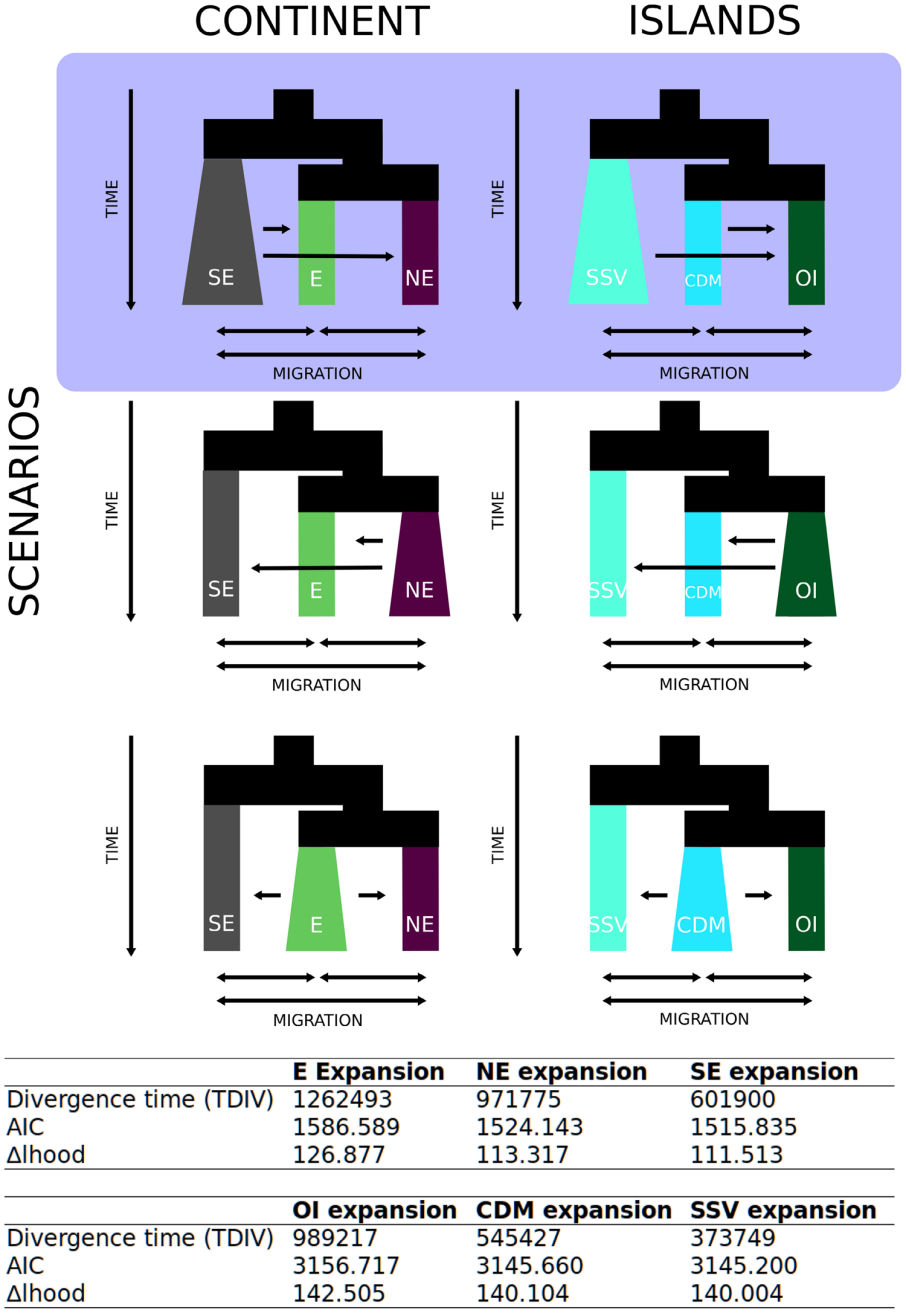
Comparisons among demographic models of population expansion for ‘continent’ (E, NE and SE), and ‘islands’ (OI, CDM and SSV) for SNPs dataset. Scenarios with lower AIC are highlighted in red shadow. *E* eastern Brazil, *NE* northestern Brazil, *SE* southeastern Brazil, *CDM* Caribbean Dominica, *OI* Brazilian oceanic islands (Rocas Atoll, Fernando de Noronha and Trindade), *SSV* San Sauveur (Dominica).

For SNPs, demographic history was assessed by Fastsimcoal with three different expansion scenarios for each dataset. As a result, the most likely expansion scenario within the 'continent' occurred on SE toward E and NE regions around 601,900 generations ago with greater gene flow from the SE to NE (Fig. 4). In the 'islands' clade, the most likely scenario was the expansion of the Caribbean SSV population to the CDM and OIs, around 373,749 generations ago and migration rates showed a more robust gene flow from SSV to the OIs (Fig. 4). The historical migration between localities also inferred by Fastsimcoal resulted in highest probabilities of migrants by SINO (SE) as source and NAT (NE) as the receiver in the 'continent', and between the equatorial islands, with RA as the source and FN as the receiver in the 'islands' (Fig. 3d, Supplementary Figs. S16 and S17). The other most likely routes of migration were from CF (SE) to BOI (E), from CDM to FN, and from FN to TR (Fig. 3e, Supplementary Fig. S17).

### Climate association analyses

Ecological niche models’ (ENMs) suitability maps were built based on the 'continent' and 'islands' distribution of specimens and variables were selected a priori based on factorial analysis: distance to shore (biogeo5), concavity (biogeo7), sea surface salinity (SSS) of the saltiest month (biogeo10), annual range in SSS (biogeo11), sea surface temperature (SST) of the coldest month (biogeo14), and annual range in SST (biogeo16), as well as bathymetry because of its biological importance for benthic macroalgae distribution (Supplementary Table S5).

**Figure 5:**
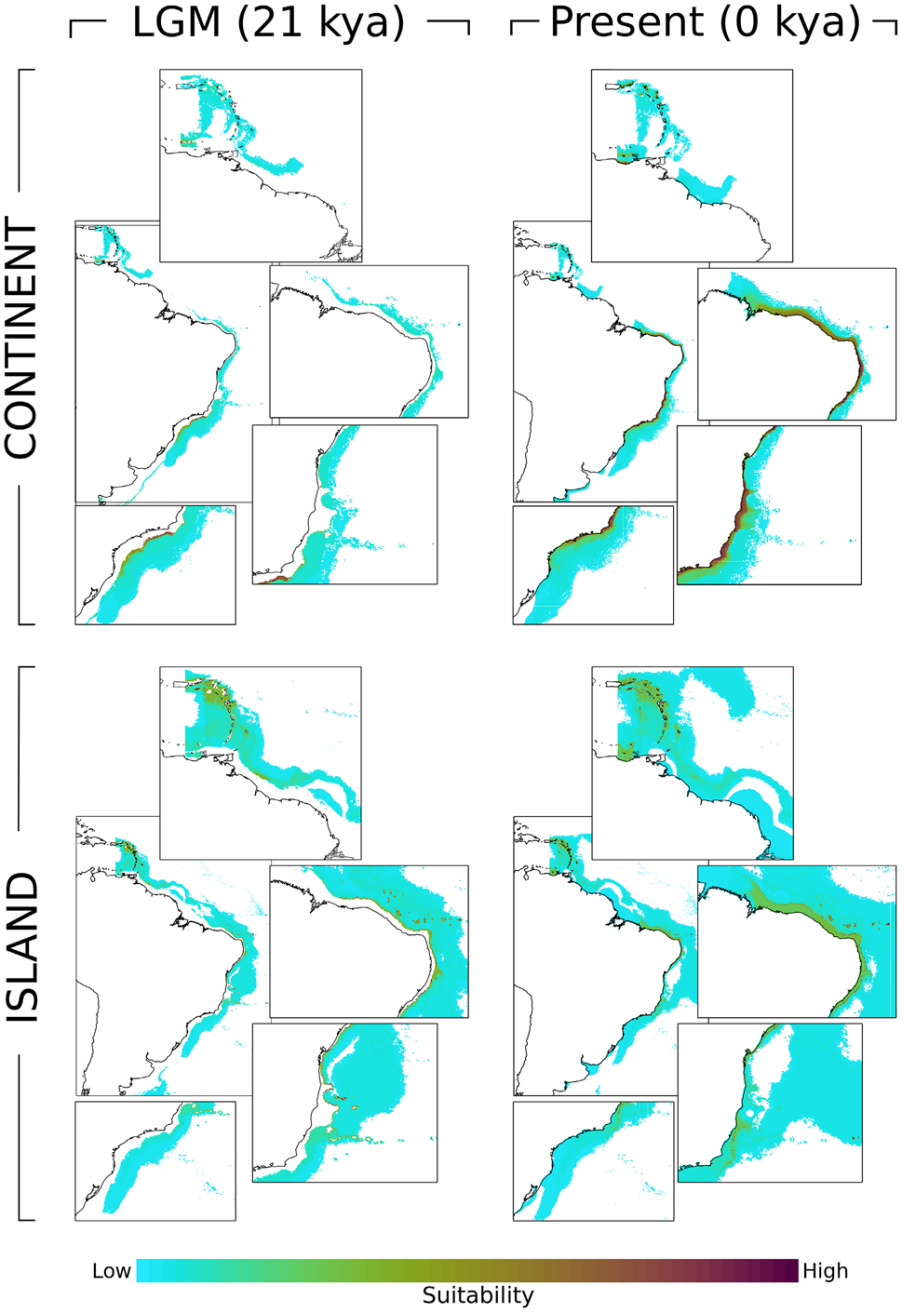
Modelled distributions of ‘continent’ and ‘islands’ for the Last Glacial Maximum (21 kya) and present (0 kya) scenarios. The suitability color range predicts how adequate the environment is for the species occurrence.

ENM results confirmed the clade 'continent' distribution suitability in the South American continent, including greater suitability on shallow waters of the continental shelf, mainly in the Brazilian SE and NE regions (Fig. 5). A small patch of adequate climate for 'continent' *A. marcuzzii* is also observed on Venezuela's coastline (Fig. 5). Suitability for 'islands' clade, on the other hand, is much lower, with a thin, suitable line on the margin of the continental shelf and islands of the FN seamount chain (Fig. 5). In the Caribbean, adequate sites include the islands of the LA (Fig. 5). Paleodistribution models suggested a considerable geographical expansion from the LGM to the present time for the 'continent' clade, with a refuge in southeastern Brazil, with higher suitability in the SE region along the margin of the continental shelf. In contrast, the paleodistribution of 'islands' revealed higher suitability in the OI, including the Vitoria-Trindade seamount chain (Fig. 5), showing a pattern of distribution retraction from the LGM to modern days. In the Caribbean, the less shallow areas in the LGM increased suitability from the past model to the present (Fig. 5).

#### SNPs

The association of environmental variables with the structure values (*F*_ST_) and geographic distance tested by the partial Mantel test for isolation-by-environment (IBE) indicated that more climatic factors are related to the 'continent' clade variation, including seafloor structure, oxygen concentration, nitrate concentration, salinity and temperature (Supplementary Table S6). On the other hand, changes in the 'islands' clade significantly varied with sea surface temperature and carbon phytoplankton biomass only (Supplementary Table S6).

**Figure 6:**
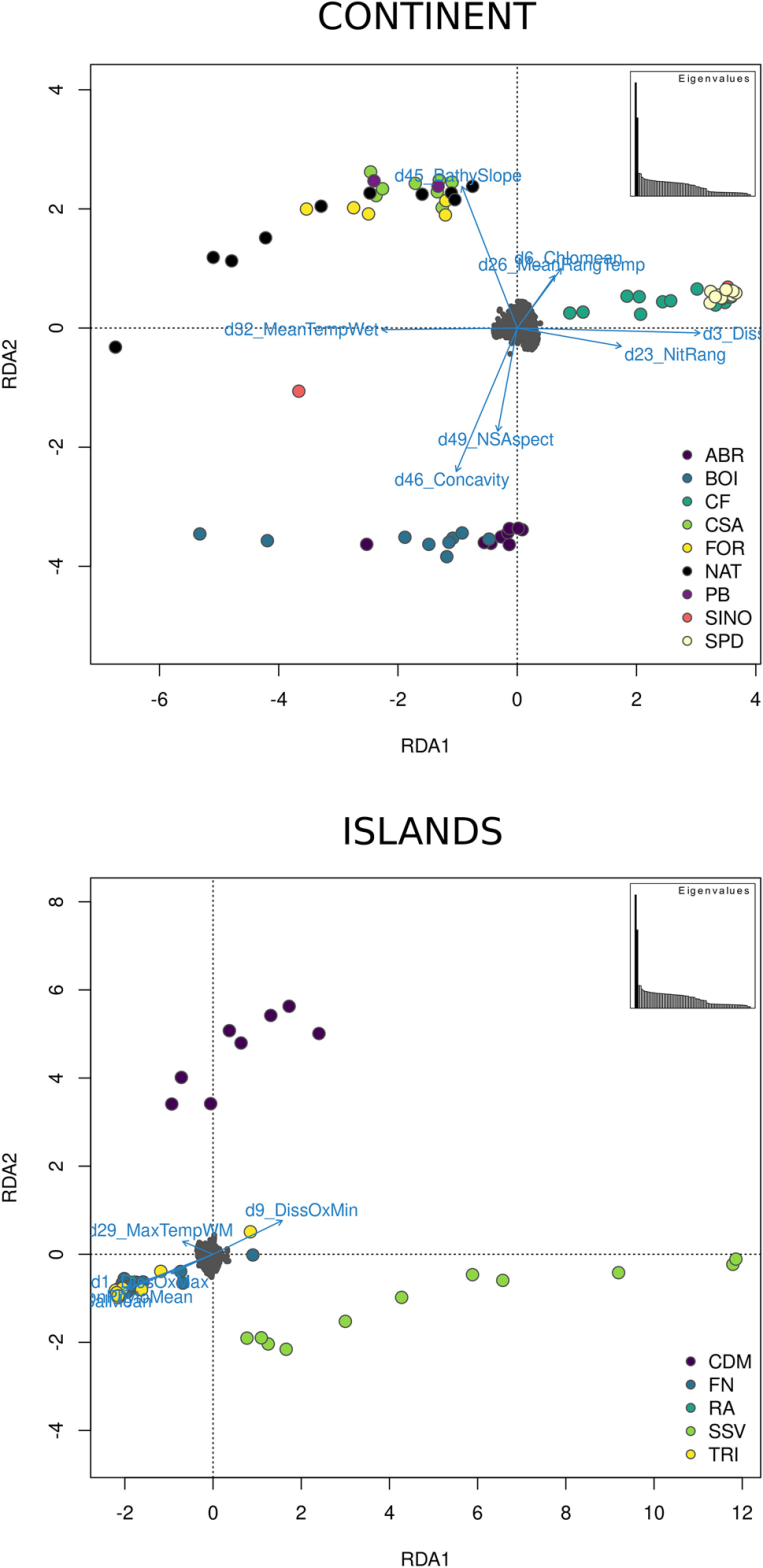
RDA results for ‘continent’ and ‘islands’ for SNPs dataset. Arrows correspond to environmental variables, and arrows length correspond to environmental variation in axis 1 and 2. Dots represent individuals and colours represent assigned localities. *ABR* Abrolhos Archipelago, *BOI* Boipeba Island, *CF* Cabo Frio, *FOR* Forte Beach, *NAT* Natal, *SINO* Ilhabela, *SPD* Ubatuba, *FN* Fernando de Noronha, *RA* Rocas Atoll, *TR* Trindade Island, *CDM* Caribbean Dominica, *SSV* San Sauveur.

The RDA recovered ten environmental variables with low correlation, with four variables common to the 'continent' and 'islands' (Supplementary Figs. S18 and S19): bathymetric slope, concavity, north/south aspect and plan curvature, all related to seafloor structure (Supplementary Table S6). Both analyses also recovered variables related to dissolved oxygen concentration, temperature and sea surface salinity, whereas chlorophyll and nitrate concentration were only found for the 'continent', and carbon phytoplankton biomass was only recovered for 'islands'. The latter two variables agree with IBE association results (Supplementary Table S6). Within the 'continent', genomic variation in SE populations of CF, SINO and SPD are explained by changes in dissolved oxygen and nitrate concentration (Fig. 6). Within the 'islands', individuals from SSV showed tremendous variation along the first axis (Fig. 6). Both Caribbean populations of SSV and CDM appear to be associated with minimum dissolved oxygen concentration, separated from each other on the second axis, while Brazilian populations from the Brazilian OIs are related to changes in maximum dissolved oxygen concentration, carbon phytoplankton biomass, sea surface salinity, annual temperature range and seafloor related variables (Fig. 6).

## Discussion

Regardless of the genome-wide and large-scale sampling, the present study only partially explain the complex evolutionary and biogeographic history of *A. marcuzzii s.l.* Two MOTUs were found widespread in the Atlantic: 'continent' and 'islands'. While the 'continent' lineage presented a clear population structure, the 'islands' presented a much more intriguing pattern, lacking IBD and with closer, highly structured populations. Despite rafting being a powerful mechanism for fauna dispersion, especially peracarid crustaceans, *A. marcuzzii s.l.* was only found associated with benthic macroalgae, being not found associated with pelagic *Sargassum* patches. Therefore, results of isolation and connectivity observed nowadays suggest complex evolutionary and biogeographic events possibly related to other traits not analyzed herein.

The genetic population structure of *Ampithoe marcuzzii s.l.* unveiled a complex evolutionary history shaped by the environment. Its direct development plays a crucial role in the lineages diversification, demonstrated by the phylogenetic, phylogenomic and structure results that characterized the separation between the South American continent ('continent') and the clade that encompasses the Lesser Antilles and Brazilian OI ('islands'). Despite not possessing *A. marcuzzii s.l.* samples from the North Atlantic coast, sampling from the Caribbean and South Atlantic demonstrated that lineages are not shaped by geographical isolation only. Thus, further sampling in the Caribbean region is essential to understand better the biogeographical processes that *A. marcuzzii s.l.* were subjected. Here, we considered *A. marcuzzii s.l.* as a species complex, and each MOTU ('continent', 'islands' and SM) was treated separately for population analyses purposes.

These patterns of diversification between continent and oceanic islands were previously observed for other peracarid species^16,19,20^, where^19^ found high levels of intraspecific divergence and endemism for Hyalidae amphipods in the Macaronesia islands using COI sequences. Similar results were found for the three most diverse orders of Peracarida (Amphipoda, Isopoda and Tanaidacea) in the region^16,20^. According to^15^, the extinction and speciation rates have a stronger role as drivers of evolution in islands than the immigration rate for species with low dispersion capacity, where dispersion might have a larger impact in the first stages of the islands’ formation.

In the western Atlantic, pieces of evidence of the 'continent' and 'islands' separation were previously observed in the Brazilian waters for the coral *Mussismilia hispida* (Verrill, 1901)^22^, showing that fauna associated with benthic macroalgae, especially the ones with low dispersion capacity, may reflect similar patterns to sessile invertebrates^22^. In agreement with *M. hispida* results, *A. marcuzzii s.l.* also presented high gene flow between TR and EI, despite being separated by over 1800 km. On the other hand, there was a lack of gene flow between EI and geographically closer continent (NAT). This scenario could be because surface currents observed nowadays do not reflect the lineages' ancient divergences or the ancient currents. Instead, we observed that other environmental variables might shape the distribution of the 'continent' and 'islands'. While several climatic features are associated with the genomic structure in the 'continent', only two variables related to the sea surface were associated with the 'islands'. Thus, a combination of abiotic and biotic acting factors might have led the 'island' clade to persevere in the harshest environments. One could hypothesize that salinity is critical for clade diversification since the OIs are mainly deprived of freshwater sources. However, sea surface salinity was not identified as a potential genetic structure driver. Despite the 'islands' clade being in the OIs, this result is probably because the same lineage is abundant in the Caribbean region, which is rich in freshwater sources and has a strong seasonal rainfall.

Indeed, the 'islands' lineage showed greater genomic diversity within most sites than the 'continent' one, possibly due to a greater ability to adapt genetically to environmental change^23^. The resilience and competitive capacity of the 'islands'’ *A. marcuzzii* might be distinctive, considering that it is the only species observed in all sampled islands so far (T. Iwasa-Arai and S. Siqueira, pers. obs.).

After the ‘continent’ and ‘islands’ separation, clades presented independent evolutionary histories, with speciation processes and environmental factors that drove each lineage to a different scenario. The population structure also reflects the direct development of amphipods, observed within populations NE, E and SE for the ‘continent’ and OI, CDM and SSV for the ‘islands’, whose strong population structure is congruent with the IBD in the ‘continent’ and the monophyly of most localities.

A recent assessment of the Brazilian marine biogeography using a multi-taxa approach suggested five geographic bioregions (Abrolhos, N/NE, NE, SE/S and S) along the continent considering species distributions, mainly associated with sea surface temperature, water turbidity and current velocity^24^. The genetic structure observed in the ‘continent’ *A. marcuzzii* was partially congruent to three bioregions, as the MOTU was not found in the southern region and the northern region was not sampled. The assemblages analyzed by^24^ clustered the state of Bahia as a sub-bioregion within the northeast bioregion, while Abrolhos was recovered as an independent bioregion. In contrast, our analyses suggested ABR grouped with other Bahia localities.

While NE localities presented low mean genetic diversity in all markers, populations from E and SE showed higher values of population diversity. This result of high diversity within SE is congruent in most analyses, including the divergence time estimates with the first split of the SE population, the migration exchange from SE to both E and NE sites and by the estimates of historical expansion of SE around 601,900 generations ago. Therefore, SE had a larger suitable area during the LGM (Fig. 5), allowing geographic and genetic diversification. In contrast, populations from the E and NE were highly affected by the lower sea levels, a pattern observed for other marine invertebrates such as marine gastropod *Littoraria flava*^25^.

The congruence of higher diversity in higher latitudes is an unusual finding since most of the marine species, from macroalgae to corals and crustaceans, presented lower levels of genetic diversity in the southernmost regions when assessed by microsatellites and mitochondrial markers^22,26^. However, higher diversity found in southernmost sites was also observed for *L. flava* in a genome-wide study^25^, indicating that evolution evidenced by SNPs reflects more recent timescales.

Barriers to gene flow across the continent are detailed and discussed in the recent review by^27^, where the bifurcation of the offshore South Equatorial Current around Cape São Roque (NE) is the most frequent barrier with phylogeographic concordance. Other representative barriers included the Vitória-Trindade seamount chain (SE), the Cabo Frio upwelling system (SE), and the main river mouths of São Francisco (NE), Paraguaçu (NE), Jequitinhonha (E), Doce (E), and Paraíba do Sul (SE^27^). Among these barriers, two barriers related to gene flow are observed as shaping the main lineages within the ‘continent’. The first separated the SE from NE and E populations and are concordant with the Vitória-Trindade seamount chain. Hence, our data suggest that the most likely scenario was vicariance driven, with the presence of a panmictic population through the continent, followed by the posterior barrier emergence in the Quaternary^28^. In addition, migration and ENM results further demonstrate the SE region as a refuge during glacial periods, with posterior migration and niche expansion in recent times, consistent with the pattern found in other marine coastal invertebrates (e.g.^25^).

Despite the historical sea level dynamics in which the seamount chain acted as a barrier, other current environmental variables may maintain the isolation in this region. The IBE and RDA results showed that variables related to the seafloor structure play an essential role in the NE, E and SE populations structure (Supplementary Table 5). In contrast, seawater composition shaped the separation of SE populations (Supplementary Table S6, Fig. 6). In the RDA, we observed that the SE localities are directly associated with changes in dissolved oxygen and nitrate concentration (Fig. 6). Both these variables are higher in sites with robust water dynamism. Cold upwelling from the South Atlantic Central Water in this region could be vital in isolating the SE lineage^22^. The second barrier found to delimit the populations is the Paraguaçu river mouth in the Todos os Santos Bay (BA). Our study succeeded in sampling sites close to each side of the barrier (i.e., FOR and BOI), which do not share haplotypes and have significant pairwise genetic structuring (Fig. 3).

Interestingly, ABR, the furthest continental island, showed high genetic diversity and high connectivity with the Eastern populations in the continent (BOI, TRA; Supplementary Table S2, Fig. 3d). The Abrolhos Archipelago is the largest and most diverse reef complex of the South Atlantic^29^. Its species composition and abiotic characteristics suggest a unique environment that differs from all other continental Brazilian marine provinces^24^. Despite its singularity, the genetic connectivity among ABR and nearby localities in the continent for other taxa, such as the coral *M. hispida*^22^ and endosymbiotic dinoflagellates associated with *Favia gravida*^30^*,* suggests that ABR might work as a genetic diversity reservoir in the region.

The second lineage is distributed throughout the Brazilian OIs of RA, FN and TR and in Dominica in the Lesser Antilles, presenting a much more complex evolutionary history. While phylogenomic analyses recovered the monophyly of all localities but FN (Fig. 1b), concatenated phylogeny of mtDNA + 28SrRNA only recovered main clades, which includes one well-supported clade from SSV, one from CDM, and one from the OIs (Fig. 1a). Although the consensus separation of the OIs from CDM, haplotype networks of 16SrRNA showed shared haplotypes between the two localities. Moreover, the structure analyses indicated a low level of admixture of the CDM genetic cluster in the FN population (Fig. 3c). This relationship between CDM and FN is corroborated by the historical migration analyses assessed with Fastsimcoal, which suggests a migration pathway from CDM to FN (Fig. 3d), and that could also be a sign of the ancient colonization route from the Caribbean to the OIs. Connectivity between the Caribbean and EI is also observed for the sally lightfoot crab *Grapsus grapsus*, a species restricted to the Caribbean and OIs in the Atlantic Ocean^22^.

Strong connectivity was observed among the OIs. Therefore, regardless of the distance between TR and EI, a robust gene flow is still observed across the southwestern Atlantic, which maintains the OI low genetic structure. The connectivity between TR and EI is also observed for the coral *M. hispida*^22^; however, these results strongly differed from other insular species that co-occur in the OIs, such as the crabs *G. grapsus* and *Johngarthia lagostoma*, that showed connectivity among the EIs and high population structure in TR island^20,31^. Thus, despite the closer phylogenetic relationship with those crabs, the population structure of the ‘islands’ amphipods agrees more with sessile invertebrates.

Dominican populations also showed an interesting pattern. The localities of the Caribbean coast (SB and SOU) displayed high connectivity, while the Atlantic coast (SSV) remained isolated. Dominica is a small island of 750 km^2^; therefore, we did not expect a high differentiation between Atlantic (SSV) and Caribbean (CDM) populations, which are closer than the Brazilian OIs. Indeed, the Structure analysis of the ‘islands’ separated into three genetic clusters, OI, CDM and SSV, in which CDM has a light contribution to the OIs’ population. PCA also showed the separation of the same three clusters, with increased differentiation of the CDM from SSV in the second axis (Fig. 3b).

SSV divergence was corroborated by most of the analyses, and demographic analyses suggest a historical expansion of SSV around 373,749 generations ago (Fig. 4), with migration to the OIs and CDM, in agreement with the species originating in the Caribbean region and later dispersing to the southwestern Atlantic. The particularities of the SSV population are better observed with the environmental association provided by the RDA, where SSV specimens presented an association with minimum dissolved oxygen concentrations (Fig. 6). It is plausible that the locality is subjected to more environmental changes than the other sampled localities within the ‘islands’ since pelagic *Sargassum* patches are regularly washed to the region during boreal summer/autumn seasons (Supplementary Fig. S20). Gases (e.g., H_2_S, NH_3_, CO_2_, CH_4_), organic matter, and high biological oxygen demand areas are formed by the biological degradation of brown algae in near-shore environments, giving rise to anaerobic zones^32,33^. In this sense, SSV is pivotal to understanding historical events and current processes that might shape future populations.

Genetic results of *Ampithoe marcuzzii s.l.* suggest that populations are geographically structured and, therefore, spatial connectivity, possibly by floating *Sargassum* dynamics, did not play a prominent role in the species' complex current pattern. Since *A. marcuzzii* is not commonly observed in floating *Sargassum*, regular *Sargassum* patches’ movements might not be enough to maintain genetic connectivity between populations on the continent. Instead, stochastic events such as seaquakes would possibly be needed to connect benthic macroalgae to the pelagic *Sargassum.* In this sense, island populations are more likely to benefit from this physical connectivity. Changes in floating *Sargassum* dynamics are yet to be understood and further studied, and future assessments of *A. marcuzzii* populations could help track the genomic changes caused by the environment.

In conclusion, marine invertebrates with limited dispersion capacities, particularly those associated with macroalgae and other benthic substrata, may exhibit similar patterns of cryptic species complexes due to isolation and abiotic variation. Based on the findings from *A. marcuzzii*, populations on the continent seem to be linked to a more dynamic range of abiotic variables and hence might be more susceptible to local climate changes, unless the continent MOTU evolves quickly to local environments, and might be less susceptible to climate changes. In contrast, island populations may be more resistant to these changes and more adaptable to changing environments. Finally, coupling the use of multiple analyses and lines of evidence to evaluate the evolutionary history of *A. marcuzzii* might help to elucidate congruent patterns in genomic population structure of macroalgae associated fauna.

## Methods

### Study sites, sampling and morphology

**Figure 7:**
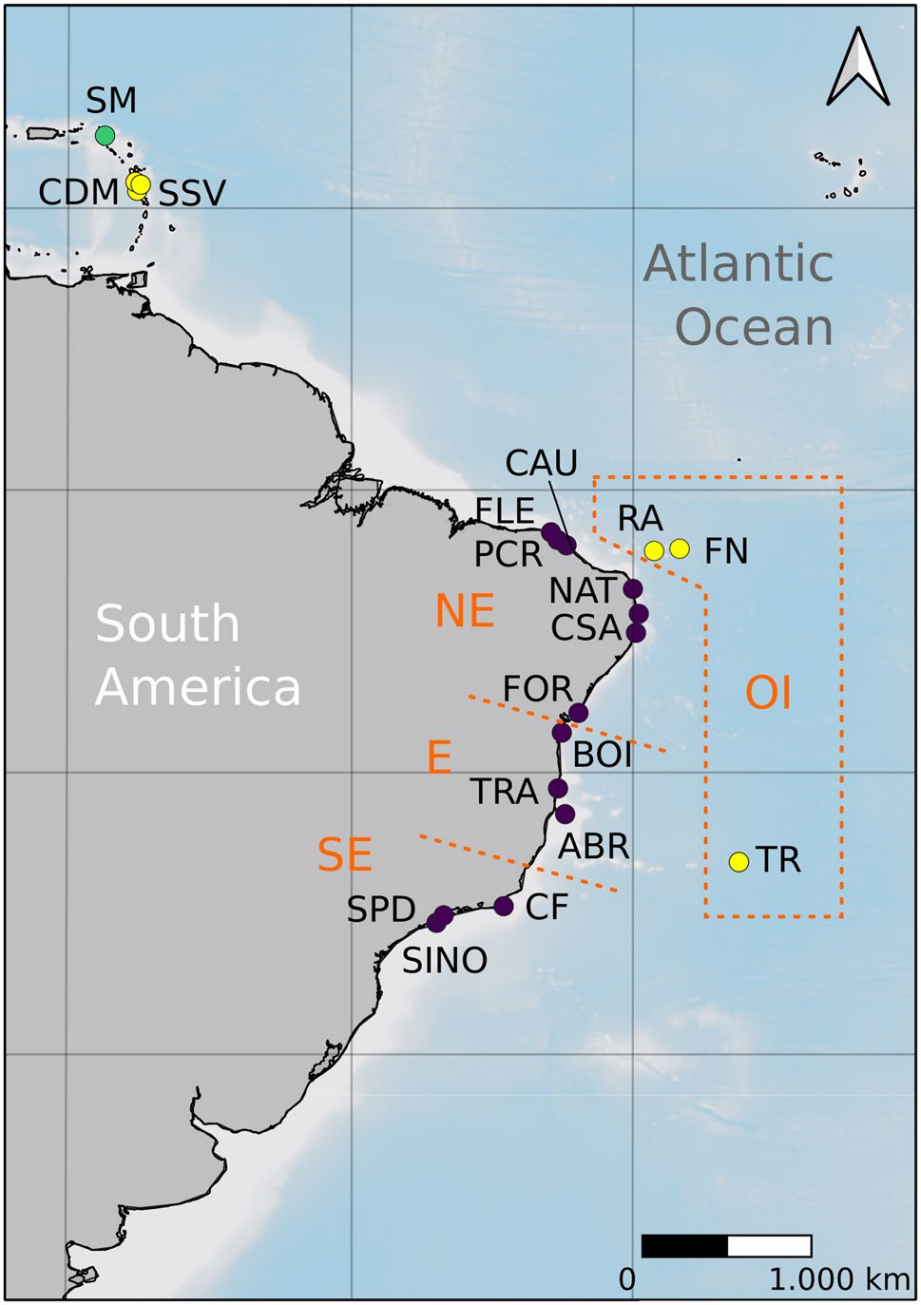
Map with sampling localities. Continent: NE: *CAU* Caucaia, *FLE* Flecheiras, *FOR* Forte Beach, *NAT* Natal, *PCR* Paracuru, E: *ABR* Abrolhos Archipelago, *BOI* Boipeba Island, *TRA* Trancoso, SE: *CF* Cabo Frio, *SINO* Ilhabela, *SPD* Ubatuba, Oceanic islands: *FN* Fernando de Noronha, *RA* Rocas Atoll, *TRI* Trindade Island. Caribbean: *SB* Secret Beach, *SM* Saint Martin, *SOU* Soufrière, *SSV* San Sauveur. Purple circles correspond to ‘continent’ localities, yellow circles correspond to ‘islands’ localities and green circle correspond to SM.

Specimens identified as *Ampithoe marcuzzii* were collected associated with macroalgae of the genera *Dictyota* J.V.Lamouroux, 1809, *Hypnea* J.V.Lamouroux, 1813 and *Sargassum* C. Agardh, 1820 between 2016 and 2021 from over 5000 km in the western Atlantic Ocean, across the northeastern (NE), eastern (E) and southeastern (SE) regions of the Brazilian coastal zone, continental and oceanic islands, and in the islands of Dominica and Saint Martin in the Lesser Antilles, Caribbean (Table 1, Fig. 7), under the Brazilian SISBIO permit no. 60924 and national authorization by Dominica's Fisheries Division.

Algal fronds were collected underwater by freediving (1–5 m) and separated from each other in fabric bags (0.2 mm mesh size). Associated fauna was fixed and preserved in 96–99% ethanol. After initial morphological sorting, specimens identified as *A. marcuzzii* were selected for molecular analysis. Identification was made based on the original description by^34^, Florida's identification guides from^11^ and comparative descriptions of the Brazilian ampithoid fauna^35^. Morphological characters used in identification included: (1) setae on uropod 1 in ascending size; (2) presence of robust setae on the ventral margin of the inner ramus of uropod 3; (3) propodus of gnathopod 2 large and rectangular; (4) size and presence of setae on antennae; (5) article 2 of mandible palp with marginal spines. Specimens and vouchers were deposited at the Museu de Zoologia da Universidade Estadual de Campinas (ZUEC CRU 4387-4408).

Sampling data is provided in Table 1. For the Brazil continent, hereafter called the 'continent', three regions were sampled: northeastern Brazil (NE), which is composed of Caucaia (CAU), Flecheiras (FLE), Forte Beach (FOR), Natal (NAT), and Paracuru (PCR); eastern Brazil (E), composed by: Abrolhos Archipelago (ABR), Boipeba Island (BOI), and Trancoso (TRA); and southeastern Brazil (SE), composed by Cabo Frio (CF), Ilhabela (SINO), and Ubatuba (SPD). The Brazilian oceanic islands' (OIs) sampling included Trindade Island (TR) and two equatorial islands (EIs), Fernando de Noronha (FN) and Rocas Atoll (RA). Sampling in the Lesser Antilles (LA) included the Caribbean Dominica (CDM), Saint Martin (SM), and San Sauveur (SSV) localities. Brazilian OIs and Dominican localities are collectively called the 'islands'.

### DNA extractions, PCR amplification and sequencing

Genomic DNA was extracted according to the CTAB protocol of^36^. Partial sequences of the mitochondrial genes cytochrome oxidase subunit 1 (COI) and 16SrRNA and nuclear 28SrRNA were obtained for subsequent analyses. Fragments were amplified using the following primer pairs: HCO2198 and LCO1490^37^ for COI, 16STf^38^ and 16Sbr^39^ for 16SrRNA and rd1 and rd4b^40^ for 28SrRNA.

Polymerase chain reactions (PCRs) were carried out in a total volume of 15μL containing 1 × PCR buffer, 2.5 U Taq DNA Polymerase, 1.5 mM MgCl_2_, 200 μM of each dNTP, 0.35 μM of each oligonucleotide and ultrapure water to achieve the reaction volume. The cycling profile on the thermal cycler involved a denaturation step at 94 °C for 1 min, 35 cycles of denaturation at 94 °C for 1 min; annealing temperature for 1 min, extension at 72 °C for 1 min; and extension at 72 °C for 10 min. Annealing temperatures were 48 °C for COI and 28SrRNA and 52 °C for 16SrRNA. PCR products were purified with polyethylene glycol solution 15% (PEG) and then amplified for sequencing using a BigDye Terminator Cycle Sequencing Kit v.3.1 (Applied Biosystems, Waltham, Massachusetts, USA) at the Human Genome and Stem Cell Research Center, Universidade de São Paulo, São Paulo, Brazil.

Chromatograms were analyzed using Genestudio 2.2 (Genestudio Inc.), which was used to perform BLAST searches (http://blast.ncbi.nlm.nih.gov) to check for contamination or sequencing errors. Individual consensus sequences were aligned using the online version of MAFFT (ver.7, see https://mafft.cbrc.jp/alignment/), with the Q-INS-i iterative refinement methods for 16SrRNA and 28SrRNA. Next, using TrimAl v.1.2^41^ with the gappyout option, we removed regions of questionable homology. Each marker was analyzed separately for most population analyses and concatenated for phylogenetic analyses.

### Library preparation, sequencing and SNP filtering

Individual libraries were generated based on the genotyping-by-sequencing (GBS) method described by^42^ and modified by^43^. Genomic DNA was digested with the *Pst*I restriction enzyme (5′-CTGCAG-3′) and ligated to barcode and common adaptors with appropriate sticky ends. The products were pooled into sets of up to 60 samples and then PCR-amplified using generic primers matching the common adaptors under the following thermocycling conditions: 5 min at 72 °C, 30 s at 98 °C, 18 cycles of 10 s at 98 °C, 30 s at 65 °C and 30 s at 72 °C, and an extension step of 5 min at 72 °C. The presence of spare adapters and the sizes of the DNA fragments were assessed by quantification on an Agilent 2100 Bioanalyzer (Agilent Technologies, Santa Clara, California, USA) with the Agilent DNA 1000 kit and by quantitative PCR using a Light Cycler 480II (Roche, Basel, Switzerland) with the Kappa Biosystems Kit (Wilmington, Massachusetts, USA) for library quantification. Libraries were constructed by EcoMol Consultoria (Piracicaba, São Paulo, Brazil), and sequencing was performed at the Center for Functional Genomics Applied to Agriculture and Agroenergy (LZT/ESALQ/USP, Brazil) in 100 bp single-end fragments using a HiSeq 2500 platform (Illumina Inc., San Diego, California, USA) in three lanes.

SeqyClean pipeline^44^ was used to filter out sequences < 50 bp and to remove sequences with an average Phred quality score ≤ 20, adapters and contaminants from the UniVec database (NCBI, ftp://ftp.ncbi.nlm.nih.gov/pub/UniVec/). The ipyrad v.0.7.28 program^45^ was used to assign reads to individual samples and to edit and cluster reads into consensus sequences through paralogue identification. In ipyrad, reads with more than five Ns or shorter than 35 bp in length were discarded. The minimum read depth was set to six for calling consensus sequences within the samples, and the maximum depth was set to 10,000. The clustering threshold was set to 90%, and the maximum number of single-nucleotide polymorphisms (SNPs) per locus was set to 30. A locus had to be present in at least 50% of the samples to be retained in the final dataset. All the remaining parameters were set as default. The density of missing data per locus was observed through matrix occupancy^46^ and PLINK^47^ removed SNPs with a minimum allele frequency of < 1%, missing genotypes higher than 50% and linkage disequilibrium (r^2^ = 0.50).

### Phylogenetic analyses and divergence time estimate

#### mtDNA and 28SrRNA

For comparative phylogenetic analyses, COI fragments, 16SrRNA and 28SrRNA fragments from *Ampithoe marcuzzii s.l.* were concatenated. Sequences of *Ampithoe ramondi* Audouin, 1826, from Brazil, were used for rooting (GenBank accession nos: OP498032, OP494671, OP494690). The best partition schemes and models were determined in ModelFinder 1.5.4^48^ based on the modified Akaike Information Criterion (AICc) available on IQ-TREE 2 web server^49^. The optimal partitioning strategy and evolutionary models consisted of subdividing the COI data set by codon positions with the model TN + F, Tne and F81 + F for each codon position, TPM3 + F + I for 16SrRNA and K2P + G4 for 28SrRNA. The phylogenetic analyses of maximum likelihood (ML) were held at the W-IQ-TREE web server^50^, and the support of the nodes was evaluated with 1000 ultrafast bootstrap replicates^51^. For the species confirmation, we performed the Automatic Barcode Gap Discovery (ABGD^52^) and the multi-rate Poisson Tree Process (mPTP^53^) on the COI dataset. Our alignments were processed in the ABGD web (https://bioinfo.mnhn.fr/abi/public/abgd/abgdweb.html) using the Kimura two-parameter substitution model prior for a maximum value of intraspecific divergence between 0.001 and 0.100, 15 recursive steps, and a gap width of 1.0. For the mPTP method, the species were inferred on ML trees in the program's web interface (http://mptp.h-its.org). mPTP improves upon the Poisson Tree Processes for single-locus species delimitation, where instead of all species sharing the same evolution rate (λ), as in PTP, each species branch has its own λ in the mPTP model. These heuristic species delimitation methods were chosen based on the monophyletic topology found in our gene trees and the single locus dataset used, optimizing their performance to the applications in COI dataset.

Divergence times were calculated with BEAST 1.8.4^54^ with the same evolutionary models and partitions from the phylogenetic analysis. We employed an uncorrelated relaxed clock with a lognormal distribution^55^. We used a random starting tree for the tree model, and speciation was modeled using the Birth–Death Process. Estimated divergence times were based on substitution rates of 1.773% Ma^−1^ for COI previously inferred for Amphipoda based on substitution rates and fossils^56^. The MCMC chain was run for 10^8^ iterations, with a thinning of 1,000. Effective sample sizes of parameters and convergence were checked with Tracer 1.7.1^57^ after discarding 20% of the trees as burn-in. Two independent runs were performed and yielded the same result. The resulting files were combined using LogCombiner 1.8^54^, and the maximum clade credibility tree was produced using TreeAnnotator 1.8^54^.

#### SNPs

The prospected SNPs from *A. marcuzzii s.l.* were used for the phylogenomic analysis using IQ-TREE v.1.5.6^49^, under the GTR + ASC model, with 1,000 bootstrap replicates.

### Genetic diversity and population structure

#### mtDNA and 28SrRNA

Two datasets ('continent': E, NE and SE; and 'islands': OI, CDM and SSV) were built for population analyses according to phylogenetic and species delimitation results. The number of haplotypes (N) and their frequencies, as well as haplotype (h) and nucleotide (π) diversities, were estimated using DNAsp v5^58^, and θ_S_ and θ_π_ were measured in Arlequin v.3.5^59^ for COI, 16SrRNA and 28SrRNA.

Pairwise genetic divergences between localities were estimated using unbiased *F*_ST_ estimator θ^60^ using Arlequin, and the population structure was examined through hierarchical analyses of molecular variance (AMOVA), clustering localities ('continent' and 'islands', separately) recovered by phylogenetic and population analyses. The statistical significance of estimates was evaluated using 10,000 permutations. Genealogical relationships among haplotypes were assessed through a haplotype network constructed using the TCS algorithm implemented in PopART v.1.7^61^.

#### SNPs

For the following analyses, two SNPs datasets were built following previously mentioned methods from the ipyrad steps, separating the species from the 'continent' and 'islands'. Specimens from Saint Martin were potentially considered a different species among *A. marcuzzii*'s complex and removed from further analyses. Diversity indexes θ_S_ and θ_π_ and fixation index *F*_IS_^60^ across loci were calculated using the Arlequin for SNPs. Principal components analysis (PCA) was implemented using the “*adegenet”* v2.1.10 package in R software to describe the variance among the genomic data^62^. Pairwise *F*_ST_ and hierarchical AMOVA were calculated as previously described for mtDNA and 28SrRNA.

The Structure software^63^ was used to analyze the complete SNP dataset for *A. marcuzzii s.l.* and the 'continent' and 'islands' datasets separately, using K values ranging from 1 to 10 according to phylogenetic and population analyses results. For each analysis, the λ value was first estimated in one run with all other values fixed, and estimated values were used in the posterior ten runs. The results from the ten iterations were combined and analyzed in the R package Structure Harvester^64^ for K likelihood comparison. The selected K was used to generate a bar plot showing the most likely ancestry of each individual in the R package “PopHelper” v2.3.1^65^.

A first Mantel test^66^ was performed to identify a possible correlation between the geographical and genetic distances between populations (IBD,^67^) using the R package "*vegan"* v2.6-4^68^. Each sampling site was considered a different population, and the genetic distance was inferred using pairwise *F*_ST_^60^. The smallest linear distance between the two sites was calculated using a latitude/longitude distance calculator provided by the National Oceanic and Atmospheric Administration website (NOAA, https://www.nhc.noaa.gov/gccalc.shtml).

### Demographic history and divergence times

#### mtDNA

Demographic processes over time were assessed for each lineage using Tajima's D^69^ and Fu's FS^70^ neutrality tests performed by Arlequin with 10,000 permutations for mtDNA. Changes in effective population sizes of each lineage over evolutionary time were inferred for mtDNA using BEAST^54^ with the same evolutionary models and partitions from the phylogenetic analysis. Analyses were run for 5,000,000 generations, with the first 500,000 generations discarded as burn-in. Convergence was detected based on sufficiently large effective sample sizes (> 200) for each parameter using Tracer 1.7^57^ and posteriorly used to plot reconstructed Bayesian skyline plots^71^.

#### SNPs

A migration matrix and demographic scenarios were estimated in Fastsimcoal v.2.7^71^ using SNPs datasets of the 'continent' and 'islands'. The historical migration rates were calculated using each locality as a population, with ten independent replicates, each including 40 estimation loops with 60,000 coalescence simulations and assuming current migration between all pairs of populations. For the population expansion scenarios, the three clusters identified in the Structure analyses were used as populations for each dataset (continent: NE, E and SE; islands: CDM, OI and SSV), and three expansion models were tested. For each model, 50 independent replicates, each including 40 estimation loops with 300,000 coalescence simulations, were performed. The probability of each model given the observed data was determined based on the maximum likelihood value and AIC. In both cases, the mutation rate was also estimated by Fastsimcoal, with prior ranging from 1 × 10^–9^ to1 × 10^–6^.

### Climate association analyses

Suitability maps were constructed under ecological niche modeling approaches (ENMs) using climate and paleoclimate simulations layers, accessed from the MARSPEC database^72^ for current and past (last glacial maximum, LGM) scenarios with a five arc min (~ 10 km resolution in the Equator region) for each lineage ('continent' and 'islands') separately to estimate the current distribution of each *A. marcuzzii*'s lineage and project their distribution in past scenarios. Climatic layers were clipped to the Western Atlantic Ocean with an extent of 80° and 20° W longitude and 35° S and 20° N latitude. Factorial analysis with Varimax was performed a priori to select variables, according to^73^.

ENMs were built using the occurrence points obtained from our sampling sites and selected climatic variables for the current climate scenario and predicted for the LGM, with the implementation of five mathematical algorithms to increase the reliability of models based on a forecast ensemble approach^74^, including three based only on the present records: (1) envelope score, Bioclim^75^; (2) Mahalanobis distance^76^; (3) Domain–Gower distance^77^; and two machine-learning methods based on presence/background records: (4) support vector machines (SVMs)^78^ and (5) maximum entropy (MaxEnt)^79^. A consensual map was built with the frequency of each grid cell predicted from all accurate models, taking values of the true skill statistic (TSS) and the "maximum sensitivity and specificity threshold" for each model^73^ using R package *“rgdal”* v1.6–7.

#### SNPs

A possible association between environmental variables and geographic distance was inferred using the isolation-by-environment (IBE) approach^80^. Mantel and partial Mantel tests were performed with 1,000 simulations. Partial Mantel tests were performed separately for each of the environmental variables, available from Bio-Oracle^81^, MARSPEC^82^ and WorldClim^83^ (Supplementary Table S7). Geographic distances were obtained using a latitude/longitude distance calculator provided by the NOAA website (https://www.nhc.noaa.gov/gccalc.shtml). The variables' distances were calculated as Euclidean distances using the dist function in R.

Lastly, the redundancy analysis (RDA) was applied to correlate the distribution and genomic traits of individuals with changes in abiotic variables^84^. We used the same environmental variables in the IBE inferences to perform pairwise correlation analyses and selected ten variables with the lowest correlation (|r|< 0.3, Supplementary Table S7) using R package “*vegan”* v2.6-4^68^.

### Supplementary Information

Supplementary Information.

## Data Availability

Raw sequence reads are deposited in the NCBI SRA (SUB12060604) and will be available after acceptance; Unique haplotype data are deposited to NCBI Nucleotide Database (COI: SUB12060469, 16SrRNA: SUB12060511, 28SrRNA: SUB12060520) and accession numbers will be available after acceptance; Related metadata will be found in NCBI SRA Metadata after acceptance.
