# Supplementary material for: Continent-island boundary and environment-shaped evolution in the marine amphipod Ampithoe marcuzzii complex (Crustacea: Eumalacostraca: Ampithoidae)
Source: Sci Rep. 2024 Jan 5;14:608. doi: 10.1038/s41598-023-51049-5 (PMC10770051; doi:10.1038/s41598-023-51049-5)
Supplement: Supplementary file 1 — Supplementary Information. [file 41598_2023_51049_MOESM1_ESM.pdf]

SUPPLEMENTARY INFORMATION

Table S1. Number of sequenced reads per specimen before and after iPyrad filtering steps, in the final consensus and final number of loci.

specimen	# reads before filtering	# reads passed filters	# reads in cluster	# reads in clusters passed filter of depth	# reads in consensus	# loci in assembly
ABR1	3162442	3162343	58529	30116	23584	3140
ABR172BF	7128230	7128004	84042	38955	31531	3347
ABR172M	3913288	3913168	61343	31120	25047	3177
ABRS2F14	3180198	3616999	58562	28790	22887	2964
ABRS2F15	4021893	4573119	78121	37586	29204	3257
ABRS4F	2012097	2288223	52201	21785	16180	2138
ABRS6CM	4126578	4692151	75265	37164	30319	3294
ABRS6DM	4971249	5654075	76224	37959	30830	3295
ABRS9M	4625170	4625022	94687	36174	29618	3278
AmFBS1m	2356549	2356495	69343	29007	24992	6060
AmFBS6m	2550914	2550862	65353	29532	25217	6116
AmFBS9Am	4722849	4722731	79453	36650	31690	6408
AmFBS9Bm	2240929	2240875	60249	28625	24830	6109
BOID10M	4320946	4320844	80595	38582	28785	3129
BOID10M27	1804519	2052227	46588	23654	18934	2754
BOID9F25	1351654	1537242	41924	18438	12875	1575
BOID9F26	771985	877957	28995	12377	8955	1065
BOIS18AM	1311746	1311699	66414	22964	18529	2532
BOIS18BM	1225887	1225846	64910	22785	18570	2400
BOIS1AF	1422470	1422434	64743	25007	20261	2738
BOIS1BF	1449776	1449733	52104	23153	18730	2769
BOIS1F28	2807578	3192671	56307	28924	23039	3024
CFS10AM	1981530	2253099	75654	29575	25393	3204
CFS10BM	1546349	1757572	68537	25589	22281	2950
CFS4AM	972545	1106226	40851	17497	15245	2362
CFS4BM	1546834	1759298	67930	24937	21589	3066
CFS4DM	2636723	2998996	62854	28748	24891	3525
CFS4F	1349187	1534652	54856	21775	18836	2859
CFS5F	1592523	1811259	66890	30869	26820	2674
CFS5M	997834	1134395	43405	18168	15804	2468
CFS6F	4954151	5633888	80899	36028	31455	3774
CFS6F44	4791894	5450119	68761	33884	29026	3678
CFS6M	3932255	4472028	75467	33514	29266	3702
CFS8F36	4664162	5303411	88072	36632	30533	3626
CFS8F41	5452728	6201208	118556	44591	37068	3730
CFS8M35	3433145	3904370	71624	28947	23585	2980
CFS8M38	4753662	5405522	82674	35689	30822	3756
CONS1M	2524117	2524059	68094	30013	24870	5960
CONS2AM	2153112	2153054	82691	33915	28438	5996
CONS2BM	1736816	1736766	71027	31575	26463	5918
CONS4	1372594	1372561	49122	22058	17413	4386
CONS5AF	3977328	3977222	78034	35726	29818	6266
CONS5BF	4095103	4095003	79621	35920	29625	6271
CONS6BF	3673951	3673841	80384	35513	29528	6230
CONS7AM	1007407	1007375	56551	22241	18548	5019
CONS7BM	5675327	5675182	106743	42976	35935	6297
CONS9	7411257	7411079	110718	53060	41325	6188
CSAD14AF	1780753	1780707	48560	23974	19133	2854
CSAD14BF	3786161	3785865	65654	31850	25563	3265
CSAD1F	3014693	3014590	65405	30723	24946	3196
CSAD1M	2136215	2136150	115896	33402	27730	3050
CSAL12F	1468446	1468400	42969	20709	16614	2525
CSAMIXF	1840915	1840855	53041	25245	18841	2754
CSAMIXM	1735912	1735827	67740	28274	23132	3028
CSAS2M	1108150	1108125	45248	19983	16158	2478
FARD6AF	3657932	3657843	82562	35822	30019	6314
FARD6BF	4182262	4182170	79342	35308	30200	6384
FB2S2AM	1933995	1933942	61025	27272	23428	5990
FB2S4AF	5042102	5041975	80310	35646	30584	6400
FBS4F	2310229	2626109	56652	29783	25556	6018
FBS4M	2101068	2388367	51835	27792	23936	5867
FBS6F	4327578	4327480	84232	35215	30249	6450
FORD14M	2563955	2563878	72172	29713	24449	3125
FORD6	746750	746715	39189	16739	13107	2071
FORD7F	1205225	1205183	79365	24534	20545	2460
FORS12F	1090594	1240363	42688	20900	16861	2411

FORS4	2752082	2751990	60626	29235	23610	3133
NATD2F21	633972	721139	26181	9808	6788	793
NATD2F22	1187448	1350096	34148	16452	12402	1578
NATD2F24	2336182	2655596	43051	19847	13622	1706
NATD2M16	1242385	1411659	39430	19188	15368	2256
NATD2M23	2849380	3239072	51612	27620	22235	2955
NATD4CM	1250906	1423120	45987	22439	17605	2543
NATD4DM	805693	915332	35888	15350	12203	1823
NATD4F	5102133	5101993	80863	35443	28379	3288
NATD5F	5849134	5848962	81145	35703	28718	3359
NATD8	5873434	5873256	79147	37555	30511	3327
CSAD2CM	2173411	2470706	52668	27168	21997	2980
CSAD2DM	1166960	1327145	50847	25235	20574	2513
PORS1M	3036114	3036034	74201	34206	28694	6200
PORS3AM	4492177	4492052	90752	38762	32292	6319
PORS3BM	7698203	7697991	91489	45475	37732	6351
PORS6F	6279580	6279396	97227	45284	37306	6404
PORS7F	4447304	4447194	85652	39489	30173	6021
PORS7M	6168302	6168134	105264	45584	37918	6356
SBS3AM	1124213	1124184	62660	24867	21172	5059
SINO100A	4773303	4773151	68239	31488	27842	3741
SINO100B	3396042	3395917	67416	30387	27003	3743
SINOS10A	216453	216445	31624	7688	6666	994
SINOS10B	4231998	4231845	75982	36364	31785	3775
SMSAM	3220583	3220503	74463	36003	29864	5351
SMSCF	4041494	4041378	84119	38441	32758	5414
SOUS3F2	1131421	1286780	41990	22001	18459	4557
SOUS7AM	1278529	1453775	53541	25805	20726	4783
SOUS7BM	837564	952064	43000	20085	16670	4118
SOUS7CM	1168488	1329205	50134	22714	18833	4422
SOUSXBM	2530601	2878311	65527	33745	26746	5561
SOUSXCM	1981128	2252928	64447	30270	25604	5540
SOUSXM5	1798378	2044717	47603	23933	18886	4084
SPD100AF	3330914	3330810	58389	28132	23922	3678
SPD100BF	2943331	2943245	90068	30588	26924	3625
SPD117AF	3224199	3224087	80677	27740	24290	3507
SPD117BF	2227440	2227347	66275	25217	21817	3372
SPD39F	3404602	3404502	84281	31403	27473	3674
SPD39M	1923929	1923879	51163	23007	20180	3311
SPD50F	2368776	2368688	52834	24349	21195	3477
SPD50M	3101009	3100939	78989	28616	24572	3465
SPD95M	1259756	1259698	48674	22710	19900	3351
SPD96M	2091417	2091345	79878	26427	23311	3423
SSVS14M	312772	355765	26909	10262	8529	1980
SSVS1F13	1496223	1701069	41696	19936	15885	3323
SSVS3F9	355739	404783	19150	7214	5205	1021
SSVS3M10	2333752	2654201	47872	24554	19312	3931
SSVS5F11	344786	392188	18955	6824	5011	908
SSVS5F12	5328314	6058517	74797	38906	32522	5683
SSVS5F8	2649203	3012796	53641	28256	23664	2611
SSVS5M	1591213	1591181	66097	27757	23821	5095
SSVS9AF	3439983	3439901	81287	37041	30334	5639
SSVS9C	1110599	1262559	45797	22067	18772	4349
SSVS9D	2729287	3104257	61422	32080	27247	5322
SSVS9M	593843	593830	35954	14705	12486	3005
SUES13F	3275333	3275261	72196	35482	28069	6141
SUES8M	1251056	1251030	138269	33177	28858	5197
TRID1AM	5538739	5538581	92641	41402	36036	6446
TRID1BM	6047437	6047266	87474	40828	35256	6445
TRID2AM	3275418	3275341	90973	38426	32766	6655
TRID2BM	4755377	4755249	79530	37320	32336	6453
TRID2CM	1016997	1157682	41561	20200	17369	4443
TRID3BF	2670743	3037196	61373	31483	27297	5915
TRID3BM	1795987	2042079	54750	27823	24056	5507
TRID3CF	2992349	3402390	70921	36475	31536	6302
TRID3F	12261962	12466997	113410	54536	44758	6475
TRID3M	3144977	3144898	71800	33647	29025	6343
TRID5M	4095241	4095136	85876	37706	32706	6424

SUPPLEMENTARY INFORMATION

Table S2. Diversity indices based on mtDNA (COI and 16SrRNA), 28SrRNA, and SNPs of *Ampithoe marcuzzii s.l.* N: number of individuals; S: polymorphic sites; K: number of haplotypes; H: haplotype diversity; π: nucleotide diversity; θ_S: nucleotide differences; θπ: nucleotide diversity; F_{IS}: fixation coefficient values; SD: standard deviation. Significant values for F_{IS} are shown in bold. ABR: Abrolhos Archipelago; BOI: Boipeba Island; CAU: Caucaia; CF: Cabo Frio; FLE: Flecheiras; FOR: Forte Beach; NAT: Natal; PCR: Paracuru: SINO: Ilhabela; SPD: Ubatuba; TRA: Trancoso; FN: Fernando de Noronha; RA, Rocas Atoll; TRI, Trindade Island; CDM, Caribbean Dominica; SM, Saint Martin; SSV, San Sauveur.

	28SrRNA							SNPs			
Locality	N	S	K	H (SD)	π (SD)	θS (SD)	θπ (SD)	N	θS (SD)	θπ (SD)	F_{IS}
CONTINENT	54	25	5	0.71 (0.03)	0.02 (0.00)	0.10 (0.10)	0.12 (0.13)	73	129.95 (46.69)	118.60 (60.62)	
PCR	6	0	1	0 (0.00)	0 (0.00)	0 (0.00)	0 (0.00)				
NAT	5	0	1	0 (0.00)	0 (0.00)	0 (0.00)	0 (0.00)	11	59.18 (19.37)	48.59 (24.31)	0.059
CSA	8	1	2	0.54 (0.12)	0 (0.00)	0.39 (0.39)	0.54 (0.56)	10	202.22 (72.01)	165.86 (84.07)	-0.016
FOR	7	0	1	0 (0.00)	0 (0.00)	0 (0.00)	0 (0.00)	5	206.08 (83.15)	194.00 (102.86)	0.109
BOI	7	0	1	0 (0.000)	0 (0.00)	0 (0.00)	0 (0.00)	9	67.74 (23.67)	57.95 (29.38)	0.051
ABR	8	1	2	0.43 (0.17)	0 (0.00)	0.39 (0.39)	0.43 (0.49)	9	217.18 (75.08)	187.48 (94.22)	0.012
CF	5	0	1	0 (0.00)	0 (0.00)	0 (0.00)	0 (0.00)	15	85.07 (26.33)	90.15 (44.37)	-0.015
SPD	8	0	1	0 (0.00)	0 (0.00)	0 (0.00)	0 (0.00)	10	144.32 (48.75)	140.095 (70.047)	-0.025
SINO								4	57.85 (25.15)	64.68 (35.68)	0.011
ISLAND	36	48	4	0.73 (0.04)	0.02 (0.01)	0 (0.00)	0 (0.00)	55	282.10 (93.23)	260.24 (129.41)	
SSV	6	0	1	0 (0.00)	0 (0.00)	0 (0.00)	0 (0.00)	11	48.48 (15.65)	41.11 (20.55)	**0.382**
CDM	9	0	1	0 (0.00)	0 (0.00)	0 (0.00)	0 (0.00)	8	226.33 (80.56)	232.18 (117.54)	-0.025
FN	8	0	1	0 (0.00)	0 (0.00)	0 (0.00)	0 (0.00)	18	401.95 (135.12)	326.86 (162.94)	0.064
RA	7	0	1	0 (0.00)	0 (0.00)	0 (0.00)	0 (0.00)	7	505.51 (159.69)	409.64 (201.48)	**0.093**
TRI	6	0	1	0 (0.00)	0 (0.00)	0 (0.00)	0 (0.00)	11	228.24 (75.16)	291.39 (144.55)	-0.056

	COI							16SrRNA						
Locality	N	S	K	H (SD)	π (SD)	θS (SD)	θπ (SD)	N	S	K	H (SD)	π (SD)	θS (SD)	θπ (SD)
CONTINENT	77	36	14	0.68 (0.06)	0.01 (0.00)	1.10 (0.66)	1.50 (1.05)	62	81	7	0.56 (0.06)	0.03 (0.01)	8.89 (4.07)	12.33 (7.12)
PCR	8	3	3	0.71 (0.12)	0 (0.00)	1.16 (0.78)	3.86 (2.47)							
FLE	4	12	2	0.12 (0.20)	0.01 (0.00)	6.55 (3.86)	8.00 (5.63)							
CAU	8	0	1	0 (0.00)	0 (0.00)	0 (0.00)	0 (0.00)							
NAT	11	0	1	0 (0.00)	0 (0.00)	0 (0.00)	0 (0.00)	11	3	3	0.73 (0.07)	0 (0.00)	1.06 (0.70)	2.18 (1.49)
CSA	9	0	1	0 (0.00)	0 (0.00)	0 (0.00)	0 (0.00)	11	0	1	0 (0.00)	0 (0.00)	0 (0.00)	0.40 (0.46)
FOR	8	0	1	0 (0.00)	0 (0.00)	0 (0.00)	0 (0.00)	8	0	1	0 (0.00)	0 (0.00)	0 (0.00)	0 (0.00)
BOI	8	1	2	0.54 (0.12)	0 (0.00)	0.39 (0.39)	0.54 (0.56)	8	9	3	0.46 (0.20)	0.01 (0.00)	18.38 (7.74)	18.47 (10.13)
TRA	2	0	1	0 (0.00)	0 (0.00)	0 (0.00)	0 (0.00)	2	0	1	0 (0.00)	0 (0.00)	0 (0.00)	0 (0.00)
ABR	8	3	3	0.68 (0.12)	0 (0.00)	1.16 (0.78)	1.07 (0.90)	8	76	2	0.25 (0.18)	0.06 (0.04)	26.10 (11.93)	31.32 (17.48)
CF								6	79	2	0.60 (0.13)	0.14 (0.03)	32.85 (15.74)	57.60 (33.66)
SPD	10	8	4	0.80 (0.09)	0.01 (0.00)	2.83 (1.46)	3.02 (1.94)	10	1	2	0.20 (0.154)	0 (0.00)	0.71 (0.54)	1.000 (0.829)
ISLAND	51	24	14	0.90 (0.02)	0.01 (0.00)	0.90 (0.61)	5.62 (3.84)	54	25	5	0.71 (0.00)	0.02 (0.00)	7.75 (4.72)	10.586 (7.852)
SSV	6	2	3	0.80 (0.12)	0 (0.00)	0.88 (0.68)	1.07 (0.94)	6	0	1	0 (0.00)	0 (0.00)	0 (0.00)	0 (0.000)
CDM	12	4	4	0.79 (0.07)	0 (0.00)	1.66 (0.93)	14.55 (7.90)	12	11	57.95 (29.38)	0.76 (0.09)	0.01 (0.00)	3.64 (1.72)	3.364 (2.087)
FN	10	7	6	0.89 (0.08)	0 (0.00)	2.47 (1.31)	2.44 (1.63)	8	1	2	0.43 (0.18)	0 (0.00)	30.17 (12.85)	31.72 (17.38)
RA	13	1	2	0.46 (0.11)	0 (0.00)	0.32 (0.32)	0.462 (0.485)	12	4	5	0.79 (0.09)	0 (0.00)	1.33 (0.79)	3.379 (2.095)
TR	10	0	1	0 (0.00)	0 (0.00)	0 (0.00)	0 (0.00)	10	0	1	0 (0.00)	0 (0.00)	0 (0.00)	1.07 (0.87)

SUPPLEMENTARY INFORMATION

Table S3. Pairwise F_{ST} values. Values in bold represent p>0.05.

CONTINENT

COI

	TRA	PCR	FLE	CAU	SPD	CSA	BOI	PB	NAT	FOR	ABR
TRA	0.00000										
PCR	-0.01266	0.00000									
FLE	0.52941	**0.30435**	0.00000								
CAU	0.00000	0.28571	**0.78082**	0.00000							
SPD	**0.37063**	**0.24070**	**0.25020**	**0.56705**	0.00000						
CSA	0.00000	**0.30769**	**0.79775**	0.00000	**0.58430**	0.00000					
BOI	**0.56912**	**0.37500**	**0.41494**	**0.73214**	**0.32441**	**0.74648**	0.00000				
PB	1.00000	0.28571	0.33333	1.00000	0.20000	1.00000	0.46429	0.00000			
NAT	0.00000	**0.34621**	**0.82470**	0.00000	**0.61441**	0.00000	**0.77079**	1.00000	0.00000		
FOR	0.00000	0.28571	**0.78082**	0.00000	**0.56705**	0.00000	**0.73214**	1.00000	0.00000	0.00000	
ABR	**0.46573**	**0.30357**	**0.32605**	**0.66071**	**0.25756**	**0.67766**	**0.39286**	0.32143	**0.70662**	**0.66071**	0.00000

16SrRNA

	NAT	CSA	FOR	BOI	ABR	CF	SPD
NAT	0.00000						
CSA	**0.24000**	0.00000					
FOR	0.19208	0.00000	0.00000				
BOI	**0.28961**	**0.31919**	**0.25926**	0.00000			
ABR	**0.17747**	**0.17837**	**0.12487**	**0.05304**	0.00000		
CF	**0.54620**	**0.55880**	**0.49250**	**0.41654**	0.20713	0.00000	
SPD	**0.78233**	**0.89654**	**0.87901**	**0.44566**	**0.28543**	**0.49784**	0.00000

28SrRNA

	PCR	NAT	CSA	FOR	BOI	ABR	CF	SPD
PCR	0.00000							
NAT	0.00000	0.00000						
CSA	0.23323	0.20000	0.00000					
FOR	0.00000	0.00000	**0.26121**	0.00000				
BOI	**1.00000**	**1.00000**	0.98817	**1.00000**	0.00000			
ABR	**0.98959**	**0.98866**	**0.98022**	**0.99039**	0.12042	0.00000		
CF	**1.00000**	**1.00000**	**0.98331**	**1.00000**	**1.00000**	**0.97530**	0.00000	
SPD	**1.00000**	**1.00000**	**0.98685**	**1.00000**	**1.00000**	**0.98052**	0.00000	0.00000

SNPs

	ABR	BOI	CF	CSAPB	FOR	NAT	SINO	SPD
ABR	0.00000							
BOI	**0.12893**	0.00000						
CF	**0.74939**	**0.74209**	0.00000					
CSAPB	**0.62352**	**0.61337**	**0.81391**	0.00000				
FOR	**0.60183**	**0.58926**	**0.81815**	**0.09267**	0.00000			
NAT	**0.62142**	**0.61288**	**0.79836**	**0.04409**	**0.10526**	0.00000		
SINO	**0.67363**	**0.65775**	**0.39823**	**0.77431**	**0.78247**	**0.75478**	0.00000	
SPD	**0.74245**	**0.73479**	**0.42378**	**0.81945**	**0.82856**	**0.80044**	**0.21029**	0.00000

ISLANDS

COI

	SSV	CDM	RA	FN	TRI
SSV	0.00000				
CDM	**0.49831**	0.00000			
RA	**0.96068**	**0.45058**	0.00000		
FN	**0.88106**	**0.36655**	**0.25460**	0.00000	
TRI	**0.97699**	**0.43925**	**0.84664**	**0.23611**	0.00000

16SrRNA

	CDM	SSV	RA	FN	TRI
CDM	0.00000				
SSV	**0.38763**	0.00000			
RA	**0.18909**	**0.58162**	0.00000		
FN	**0.07360**	**0.14937**	**0.07187**	0.00000	
TRI	**0.14595**	**1.00000**	**0.19603**	0.02946	0.00000

28SrRNA

	CDM	SSV	RA	FN	TRI
CDM	0.00000				
SSV	**1.00000**	0.00000			
RA	**1.00000**	**1.00000**	0.00000		
FN	**1.00000**	**1.00000**	0.00000	0.00000	
TRI	**1.00000**	**1.00000**	**1.00000**	**1.00000**	0.00000

SNPs

Table S4. Neutrality tests results for mtDNA. Values in bold represent $p < 0.005$.

Lineage	Tajima's D	P-value D	Fu's Fs	P-value Fs
NE	-1.19488	0.10900	34.00000	1.00000
E	14.59037	1.00000	11.70793	0.99980
SE	0.08807	0.59080	14.47543	1.00000
CDM	-0.79866	0.22590	2.33347	0.84690
OI	**-2.69152**	0.00000	7.40179	0.99120
SSV	-0.27492	0.39220	10.27260	0.99930

Table S5. Variable selection results based on Factorial Analysis.

	ML1	ML2	ML3	ML4	ML5
Bathymetric slope	0.01	0.03	0	0.02	0.09
Concavity	0.17	0.06	0	0.04	0.02
Distance to shore	0.08	0.03	0.65	0	0.01
East/West aspect	0.06	0.05	0.75	0.01	0
North/South Aspect	0.23	0.18	0.01	0.09	0.48
Plan curvature	0.25	0.12	0.14	0.04	0.02
Profile curvature	0.02	0.01	1	0	0
Sea surface salinity (annual mean)	0.3	0.61	0.01	0.73	0.03
Sea surface salinity (monthly minimum)	0.1	0.88	0.01	0.44	0.14
Sea surface salinity (monthly maximum)	0.49	0.16	0	0.84	0.16
Sea surface salinity (annual range)	0.12	0.96	0.01	0.11	0.24
Sea surface salinity (annual variance)	0.04	0.84	0.01	0.09	0.12
Sea surface temperature (annual mean)	0.92	0.04	0	0.19	0.35
Sea surface temperature (coldest ice-free month)	0.95	0.04	0	0.2	0.25
Sea surface temperature (warmest ice-free month)	0.85	0.06	0	0.18	0.48
Sea surface temperature (range)	0.95	0.03	0.01	0.2	0.25
Sea surface temperature (variance)	0.84	0.04	0	0.37	0.32
Bathymetry	0.05	0.37	0.09	0.24	0.29

Table S6. Isolation-by-environment (IBE) partial Mantel environmental variables and significative ($p<0.05$) results.

	**Variable**	**Mantel r^2**	**p-value**
CONTINENT	Bathymetry	0.572	0.003
	Bathymetric slope	0.576	0.011
	Dissolved oxygen concentration (maximum at min depth)	0.520	0.020
	Dissolved oxygen concentration (mean at min depth)	0.391	0.043
	Nitrate concentration (range at min depth)	0.636	0.003
	Sea surface salinity (mean)	0.448	0.027
	Mean Temperature of Driest Quarter	0.469	0.024
ISLANDS	Sea surface temperature (warmest ice-free month)	0.620	0.033
	Carbon phytoplankton biomass (maximum at min depth)	0.702	0.017

Table S7. Climatic variables used for IBE and RDA. Environmental variables used in RDA of *Ampithoe marcuzzii s.l.* for continent (†) and islands (‡) according to minimum correlation from Bio-Oracle, World Clim and MARSPEC databases.

**Bio-Oracle Variables**	**WorlClim variables**
Dissolved oxygen concentration (maximum at min depth)‡	Annual Mean Temperature
Dissolved oxygen concentration (longterm max at min depth)	Mean Diurnal Range (Mean of monthly (max temp - min temp))
Dissolved oxygen concentration (range at min depth)†	Isothermality (BIO2/BIO7) (×100)
Chlorophyll A (maximum)	Temperature Seasonality (standard deviation ×100)
Dissolved oxygen concentration (mean at min depth)	Max Temperature of Warmest Month‡
Chlorophyll A (mean)†	Min Temperature of Coldest Month
Dissolved oxygen concentration (longterm min at min depth)	Temperature Annual Range (BIO5-BIO6)‡
Chlorophyll A (range)	Mean Temperature of Wettest Quarter†
Dissolved oxygen concentration (minimum at min depth)‡	Mean Temperature of Driest Quarter
Chlorophyll A (minimum)	Mean Temperature of Warmest Quarter
Carbon phytoplankton biomass (maximum at min depth)	Mean Temperature of Coldest Quarter
pH	Annual Precipitation
Carbon phytoplankton biomass (range at min depth)	Precipitation of Wettest Month
Sea water temperature (range at min depth)	Precipitation of Driest Month
Sea water temperature (range at sea surface)	Precipitation Seasonality (Coefficient of Variation)
Sea surface temperature (range)	Precipitation of Wettest Quarter
Carbon phytoplankton biomass (longterm max at min depth)	Precipitation of Driest Quarter
Carbon phytoplankton biomass (mean at min depth)‡	Precipitation of Warmest Quarter
Chlorophyll concentration (maximum at min depth)	Precipitation of Coldest Quarter
Chlorophyll concentration (range at min depth)	
Chlorophyll concentration (longterm max at min depth)	
Nitrate	
Nitrate concentration (range at min depth)†	
Sea surface salinity (mean)‡	
**MarSpec variables**	
Bathymetry	Sea surface salinity (monthly minimum)
Bathymetric slope†/‡	Sea surface salinity (monthly maximum)
Concavity†/‡	Sea surface salinity (annual range)†
Distance to shore	Sea surface salinity (annual variance)
East/West aspect	Sea surface temperature (annual mean)
North/South Aspect†/‡	Sea surface temperature (coldest ice-free month)
Plan curvature†/‡	Sea surface temperature (warmest ice-free month)
Profile curvature	Sea surface temperature (range)
Sea surface salinity (annual mean)	Sea surface temperature (variance)

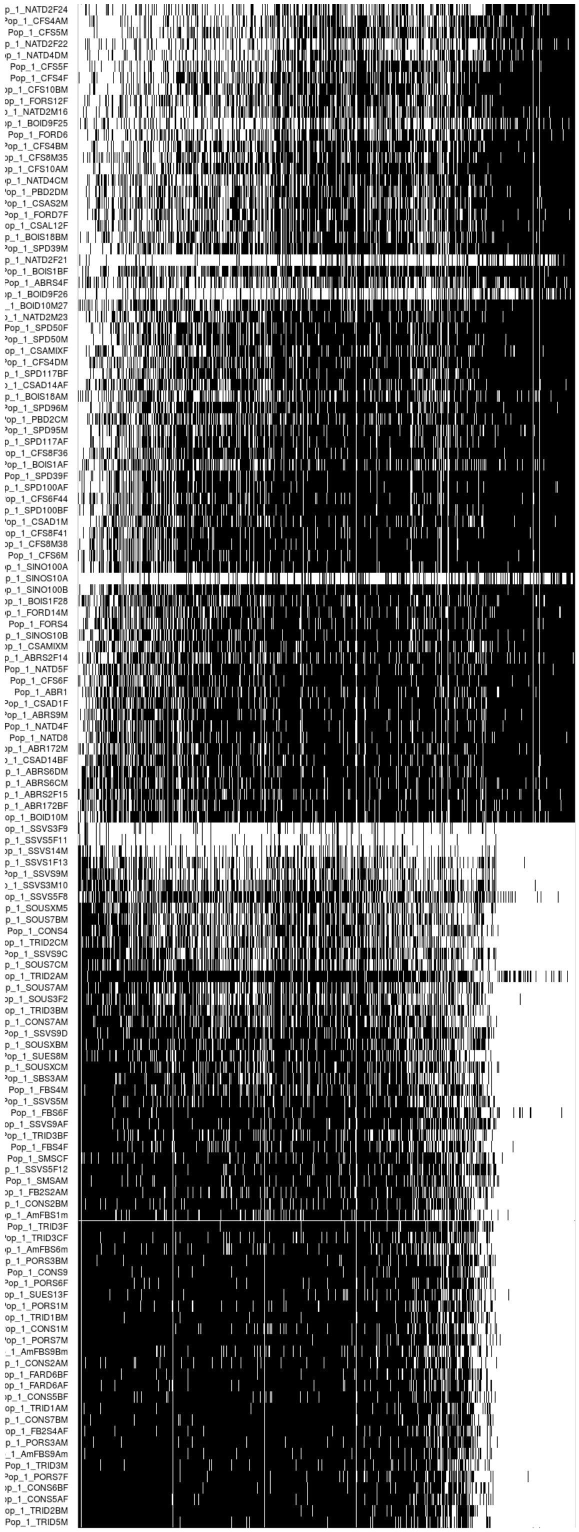

Figure S1. Matrix Condenser results showing 5,857 SNPs after PLINK filtering for ‘merged’ dataset. Each column is a SNP, where the black bars represent a SNP present in the individual, and the blank bars indicate that the SNP is missing.

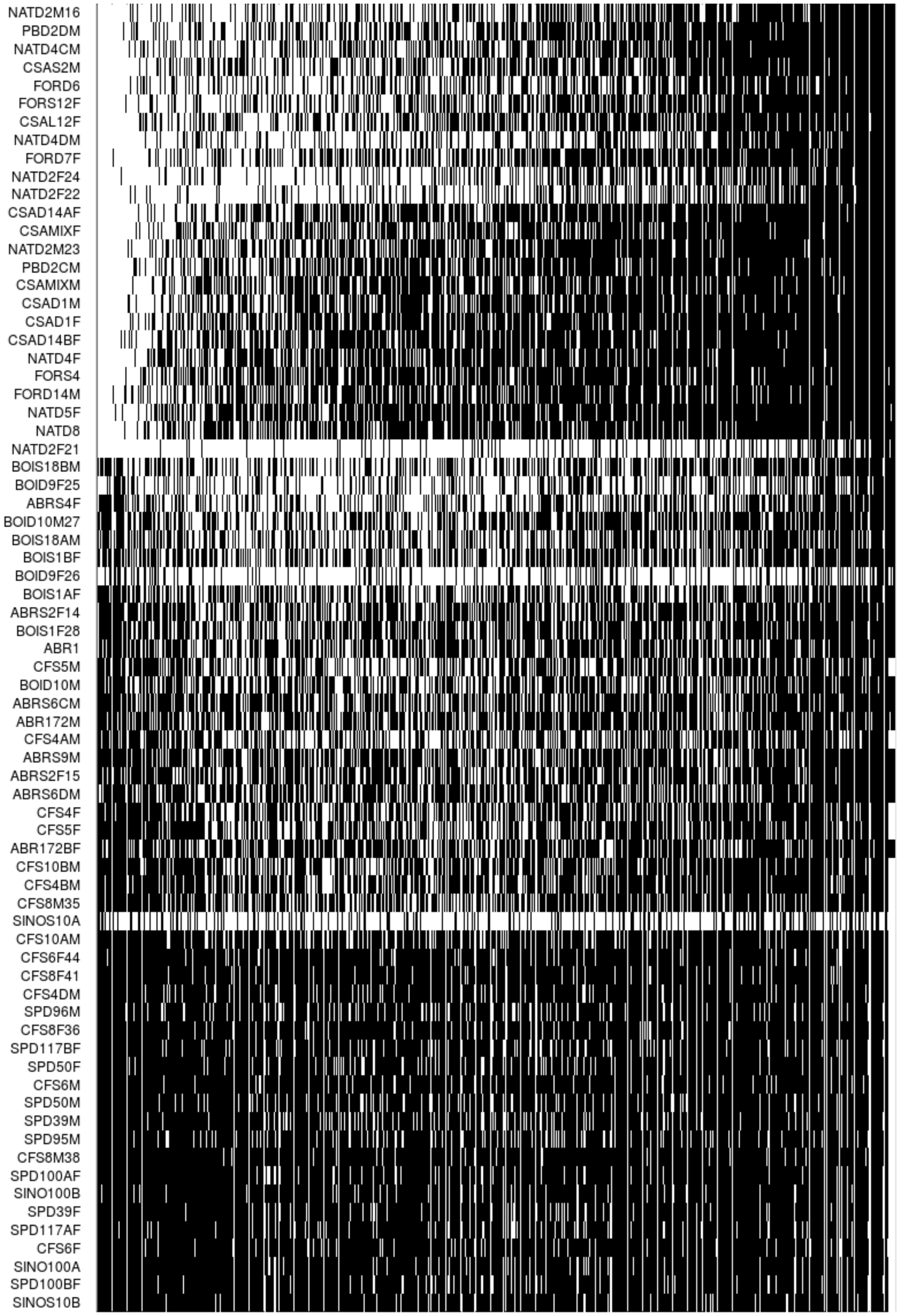

Figure S2. Matrix Condenser results showing 21,126 SNPs after PLINK filtering for ‘continent’ dataset. Each column is a SNP, where the black bars represent a SNP present in the individual, and the blank bars indicate that the SNP is missing.

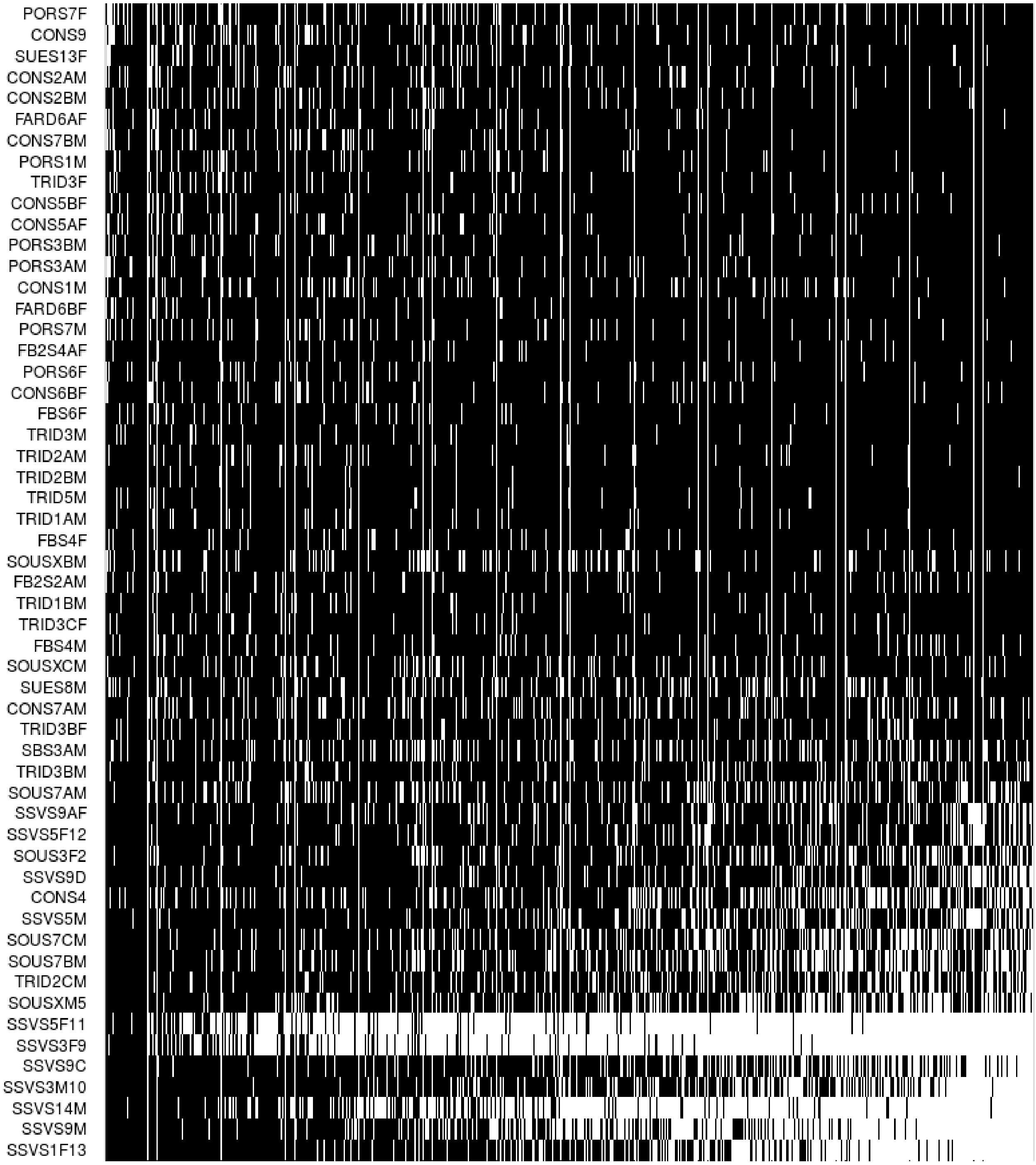

Figure S3. Matrix Condenser results showing 17,237 SNPs after PLINK filtering for ‘islands’ dataset. Each column is a SNP, where the black bars represent a SNP present in the individual, and the blank bars indicate that the SNP is missing.

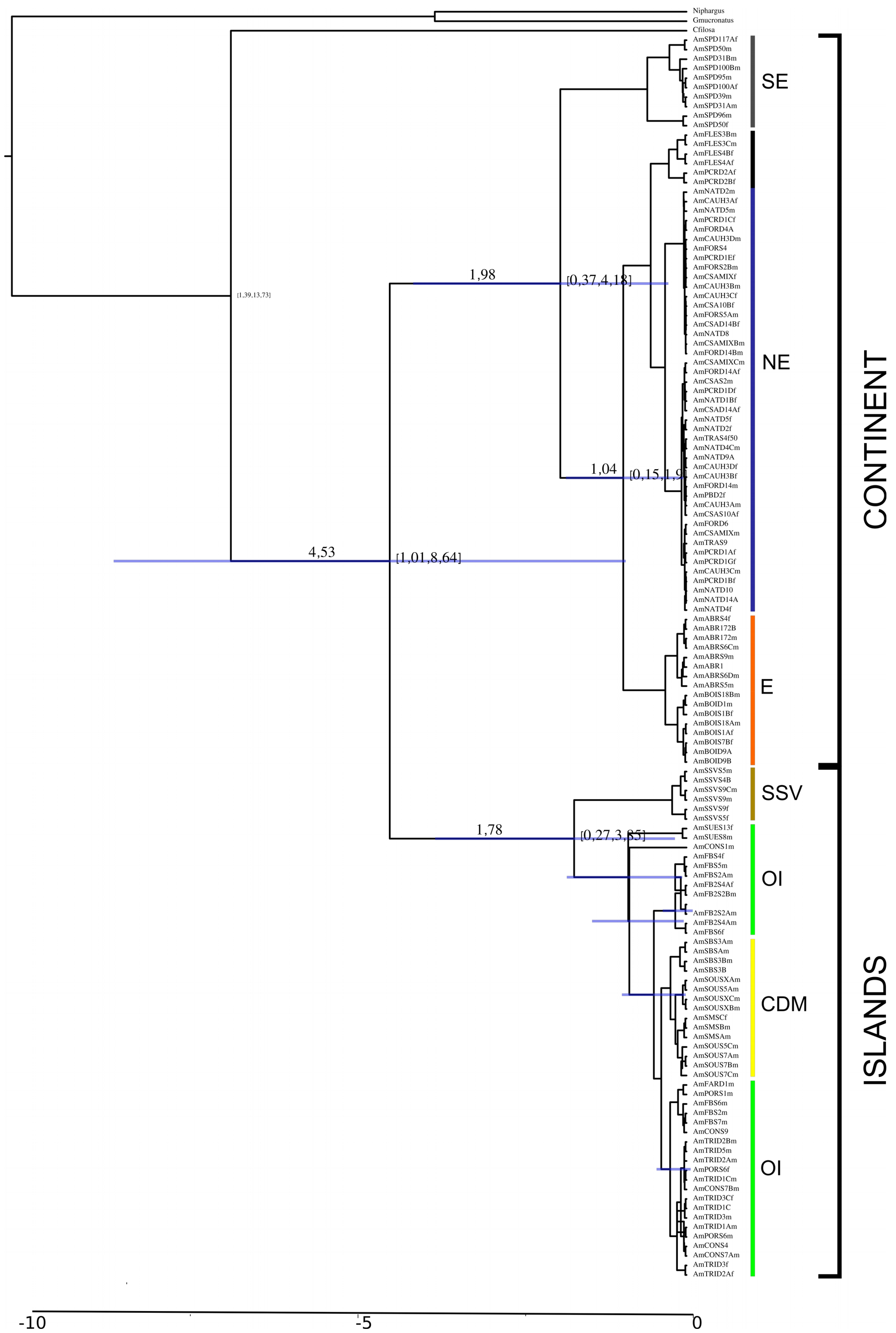

Figure S4. Bayesian calibrated tree of *Ampithoe marcuzzii s.l.* using COI Numbers above branches represent mean HPD. Node bars represent the 95% HPD interval. Time divergence bar is in a Mya scale.

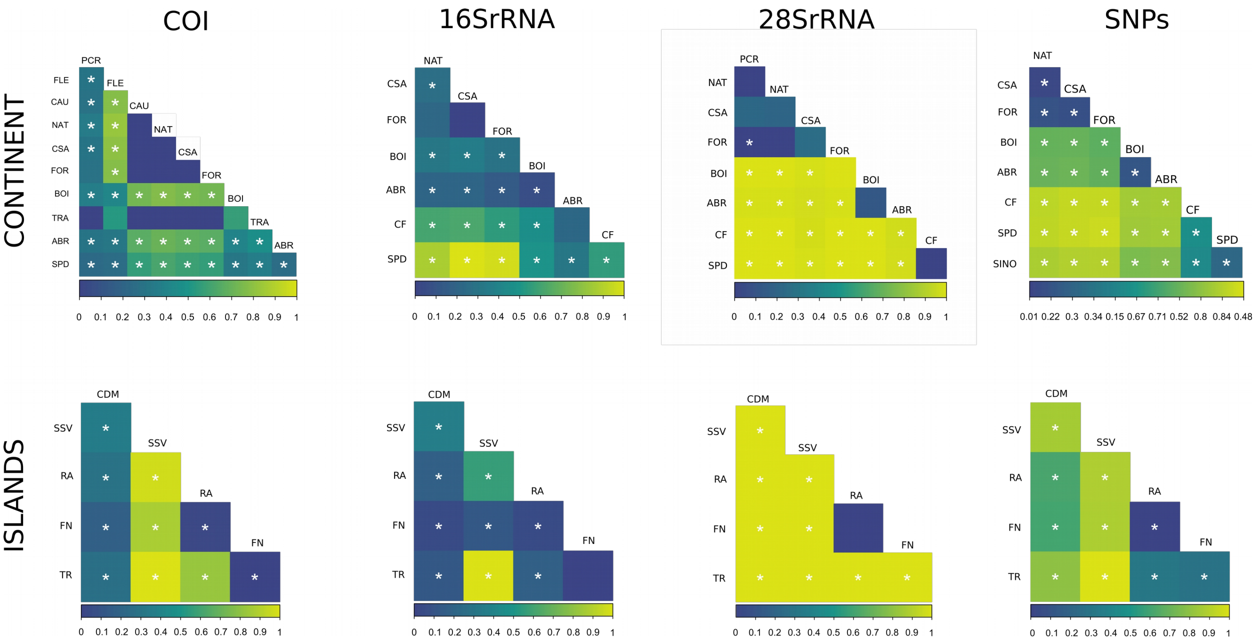

Figure S5. Pairwise F_{ST} heatmaps for each genetic marker. Colour coding illustrates the observed F_{ST} value. Asterisks correspond to significant values (P < 0.05). ABR: Abrolhos Archipelago; BOI: Boipeba Island; CAU: Caucaia; CF: Cabo Frio; FLE: Flecheiras; FOR: Forte Beach; NAT: Natal; PCR: Paracuru; SINO: Ilhabela; SPD: Ubatuba; TRA: Trancoso. FN: Fernando de Noronha; RA: Rocas Atoll; TR: Trindade Island; CDM: Caribbean Dominica; SSV, San Sauveur.

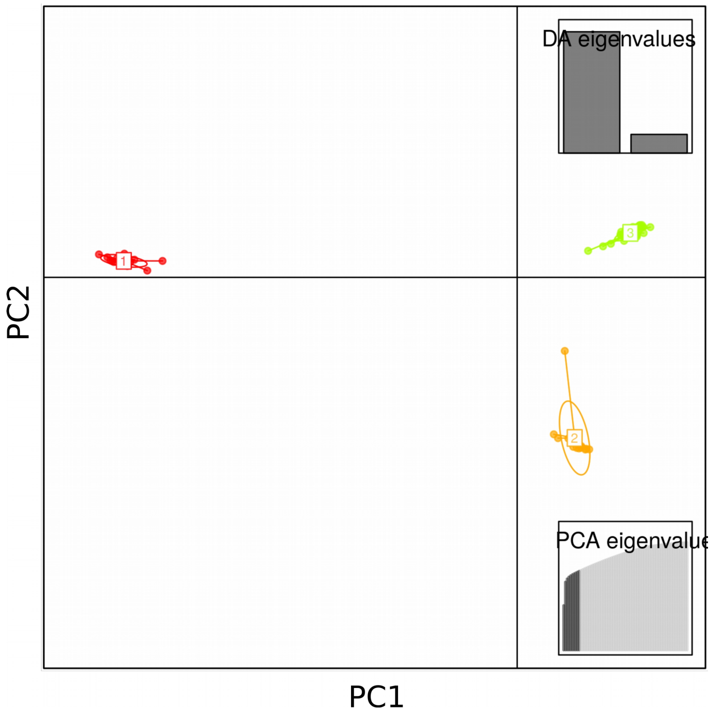

Figure S6. DAPC results for 'continent'. Cluster 1: E; Cluster 2: NE; Cluster 3: SE.

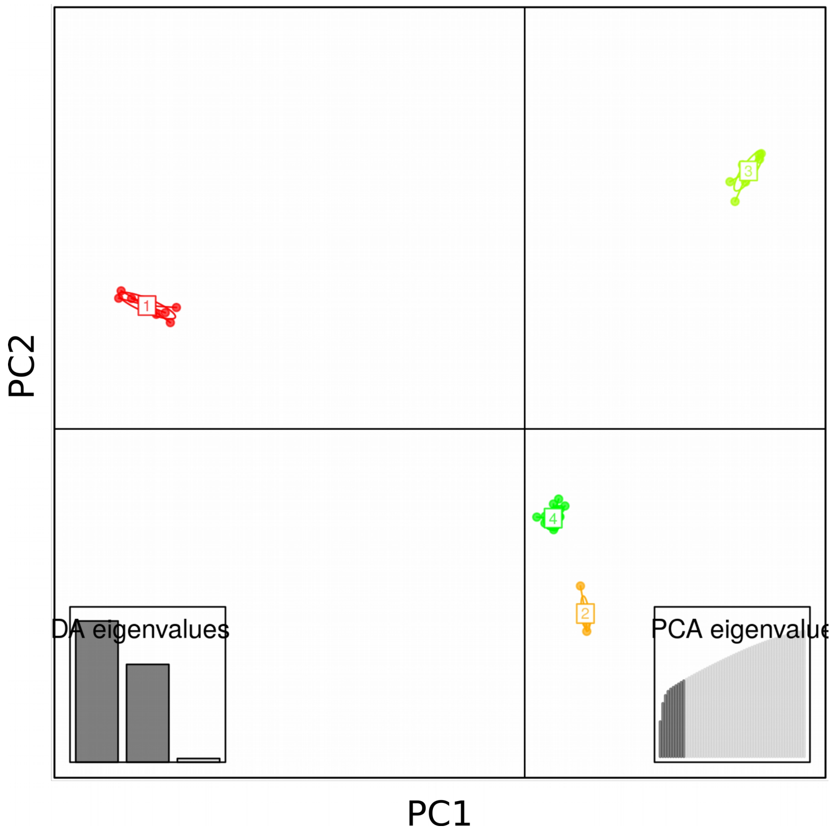

Figure S7. DAPC results for 'islands'. Cluster 1: OI; Cluster 2: CDM; Cluster 3: SSV (1); Cluster 4: SSV (2).

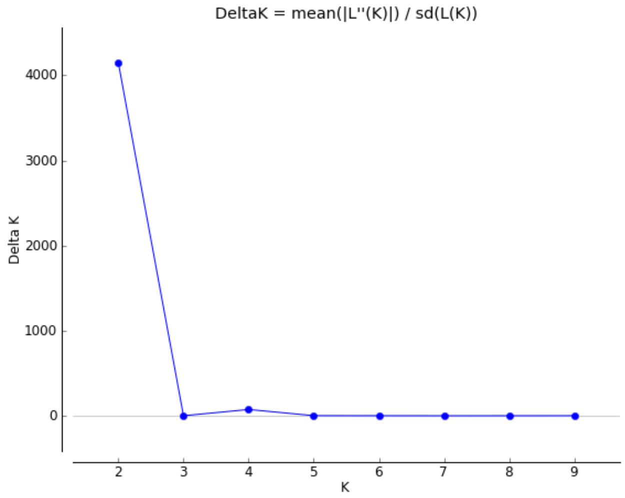

Figure S8. Structure Harverster results for each ΔK for *A. marcuzzii s.l.*

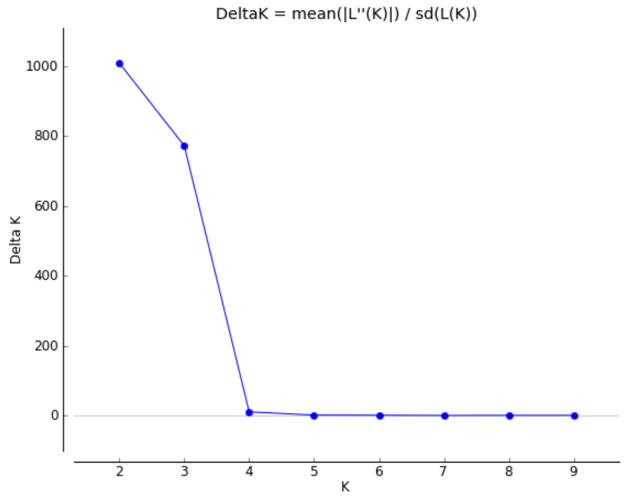

Figure S9. Structure Harverster results for each ΔK for ‘continent’.

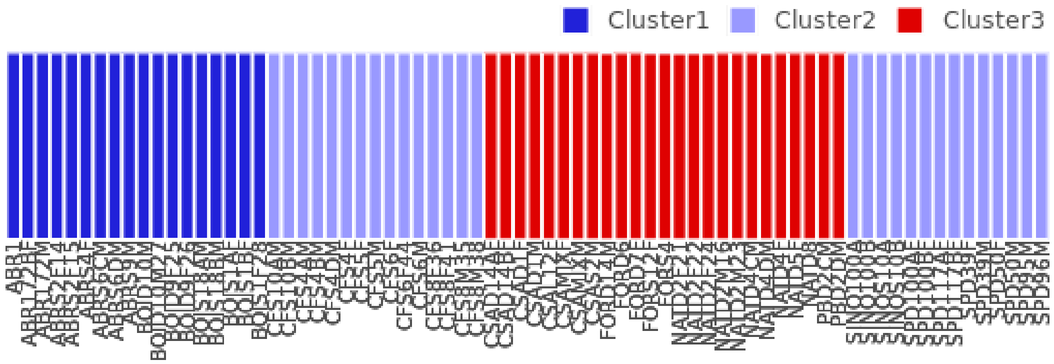

Figure S10. Structure results for ‘continent’ with second highest ΔK value of k=3.

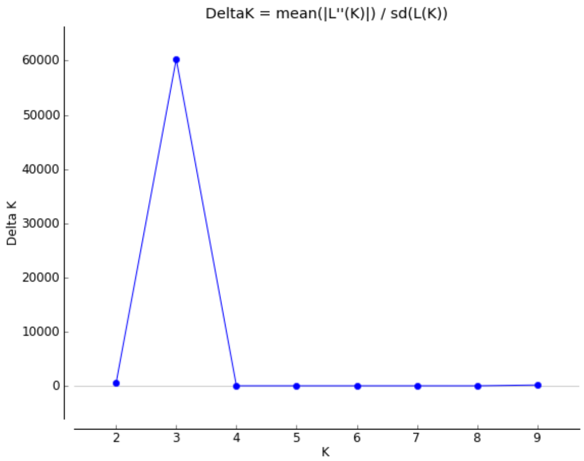

Figure S11. Structure Harverster results for each ΔK for 'islands'.

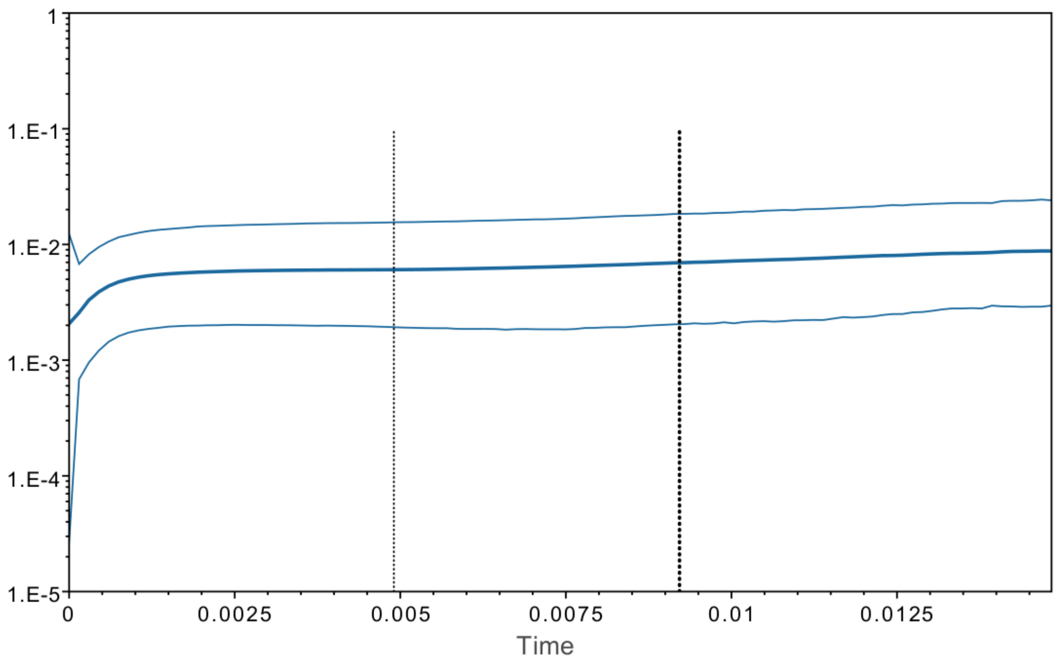

Figure S12. Bayesian Skyline Plot of E population.

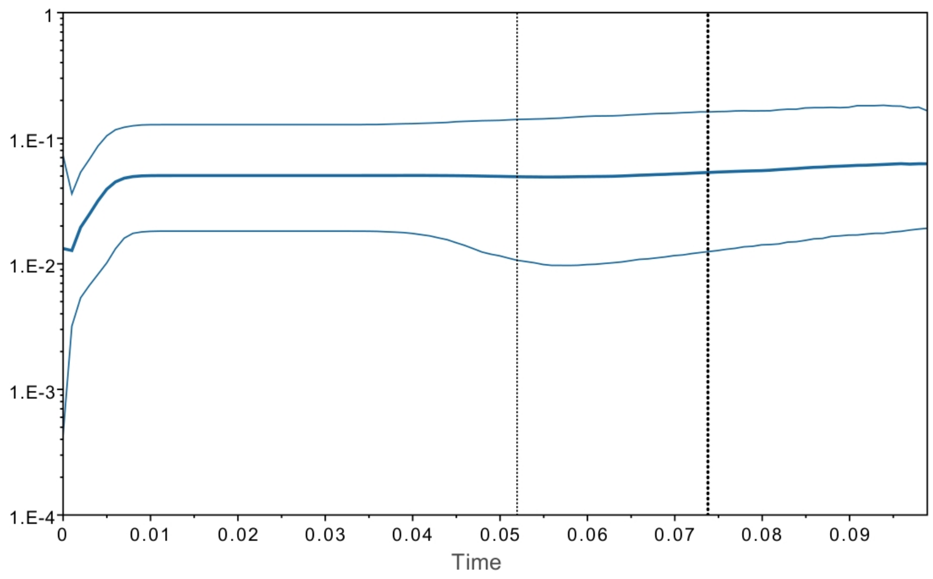

Figure S13. Bayesian Skyline Plot of NE population.

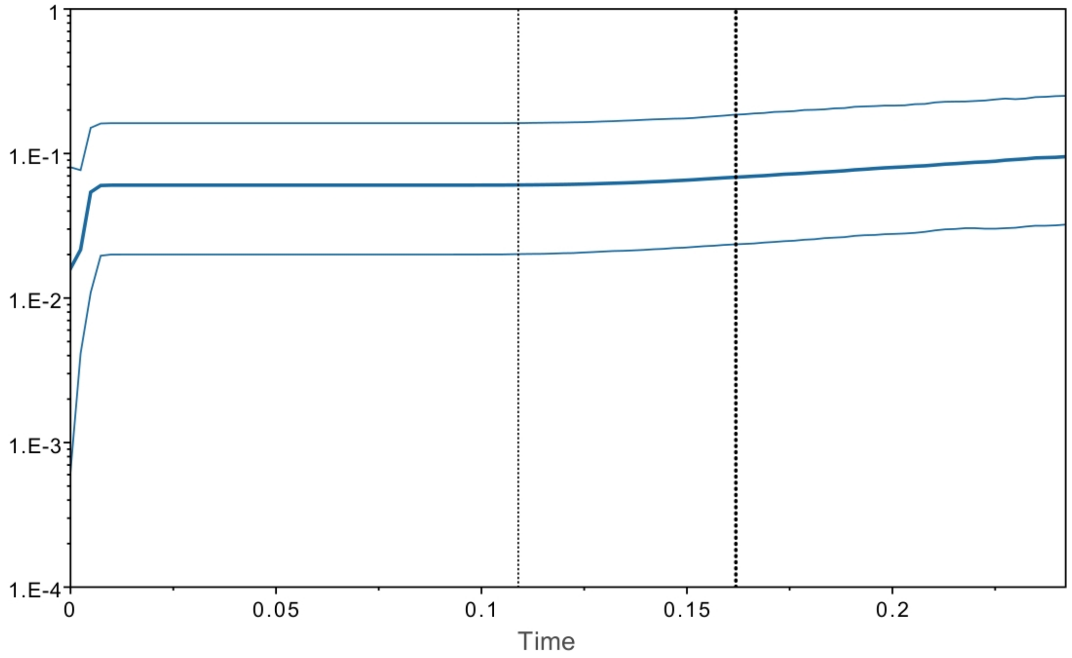

Figure S14. Bayesian Skyline Plot of SE population.

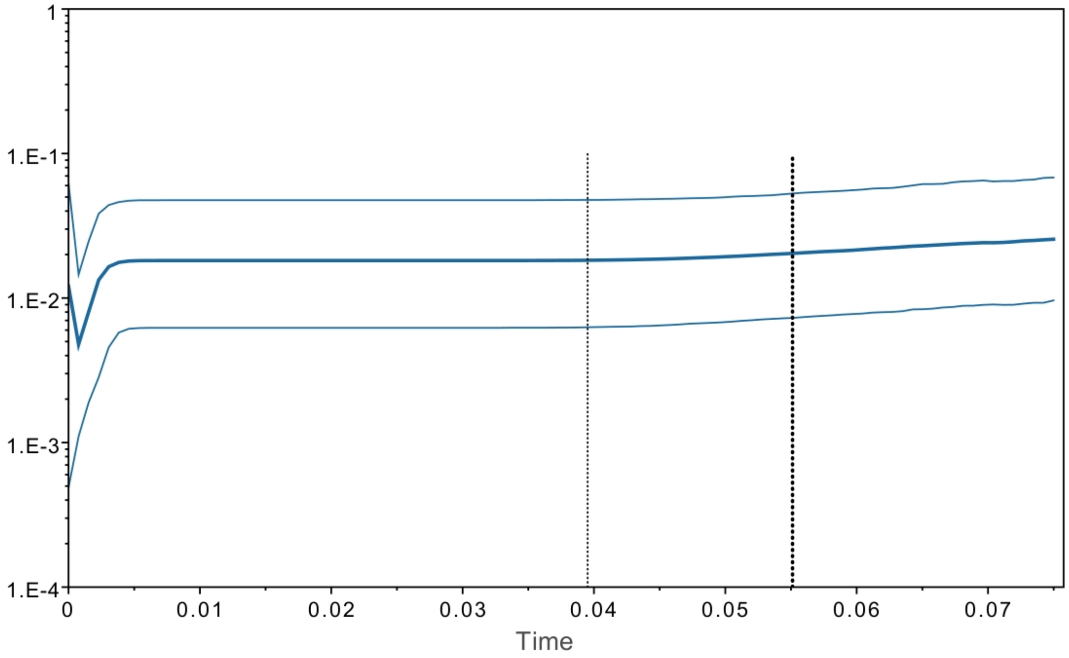

Figure S15. Bayesian Skyline Plot of OI population.

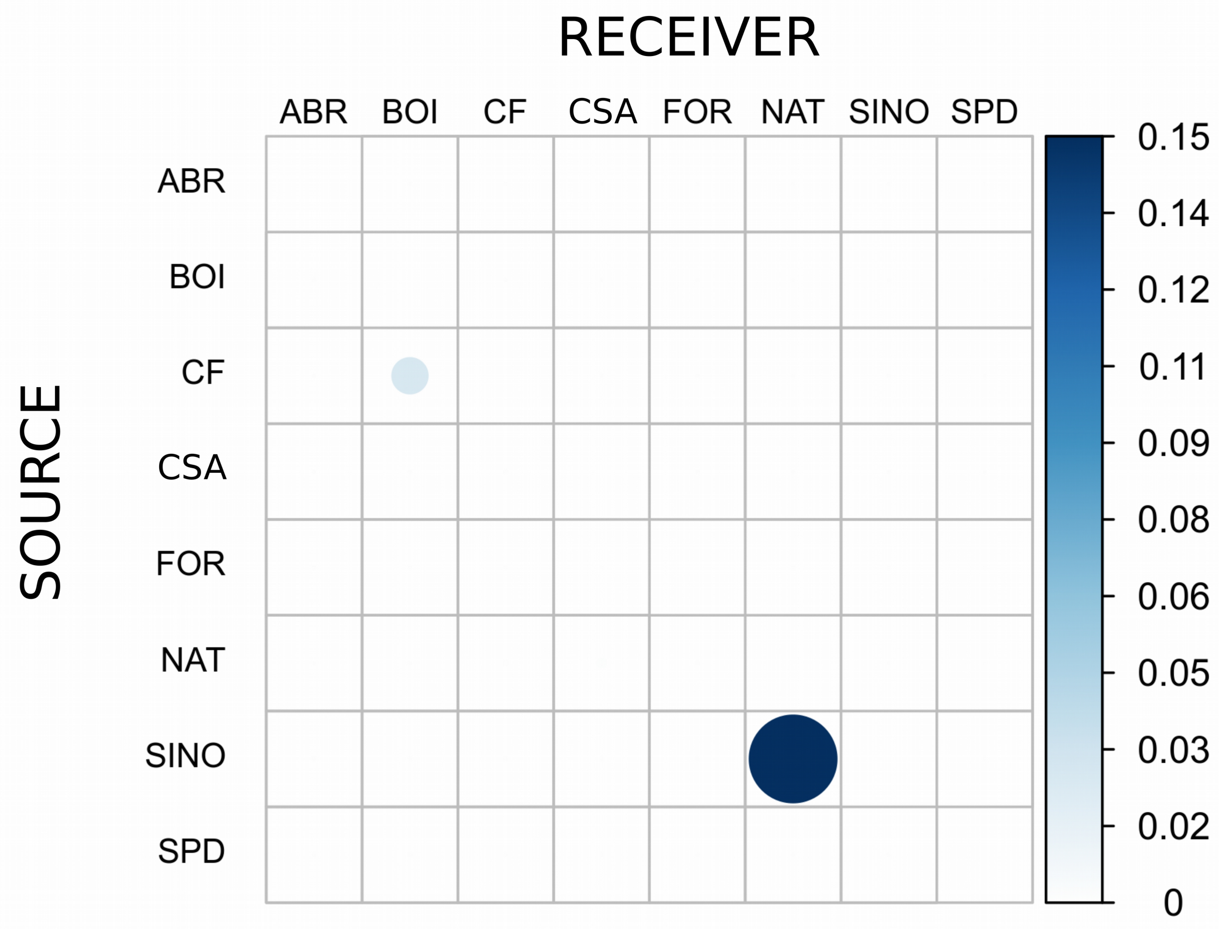

Figure S16. Fastsimcoal migration results for ‘continent’.

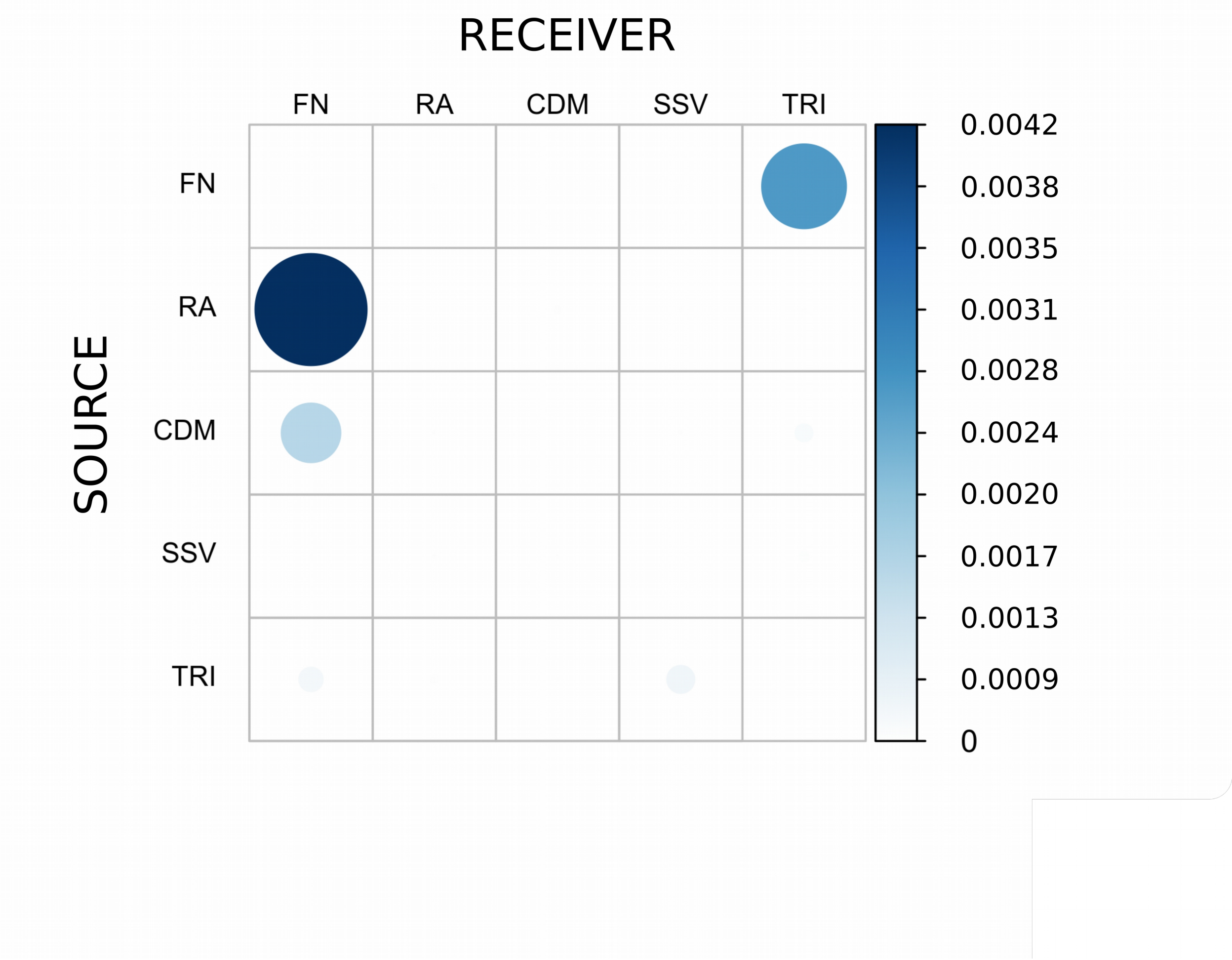

Figure S17. Fastsimcoal migration results for ‘islands’.

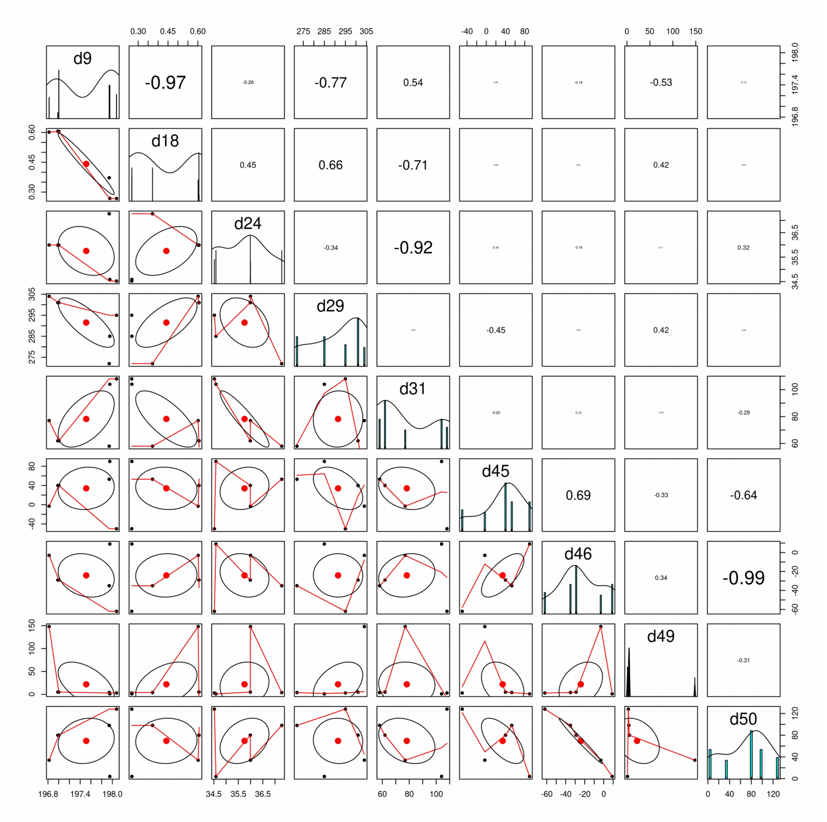

Figure S18. Pairwise correlation with ten chosen variables for ‘continent’.

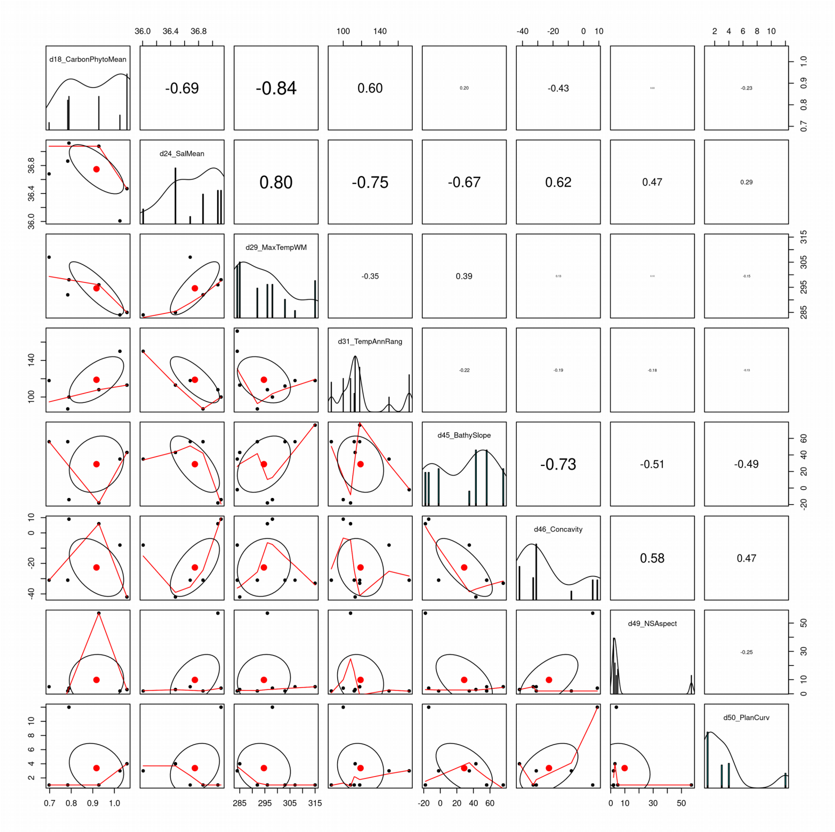

Figure S19. Pairwise correlation with ten chosen variables for ‘islands’.

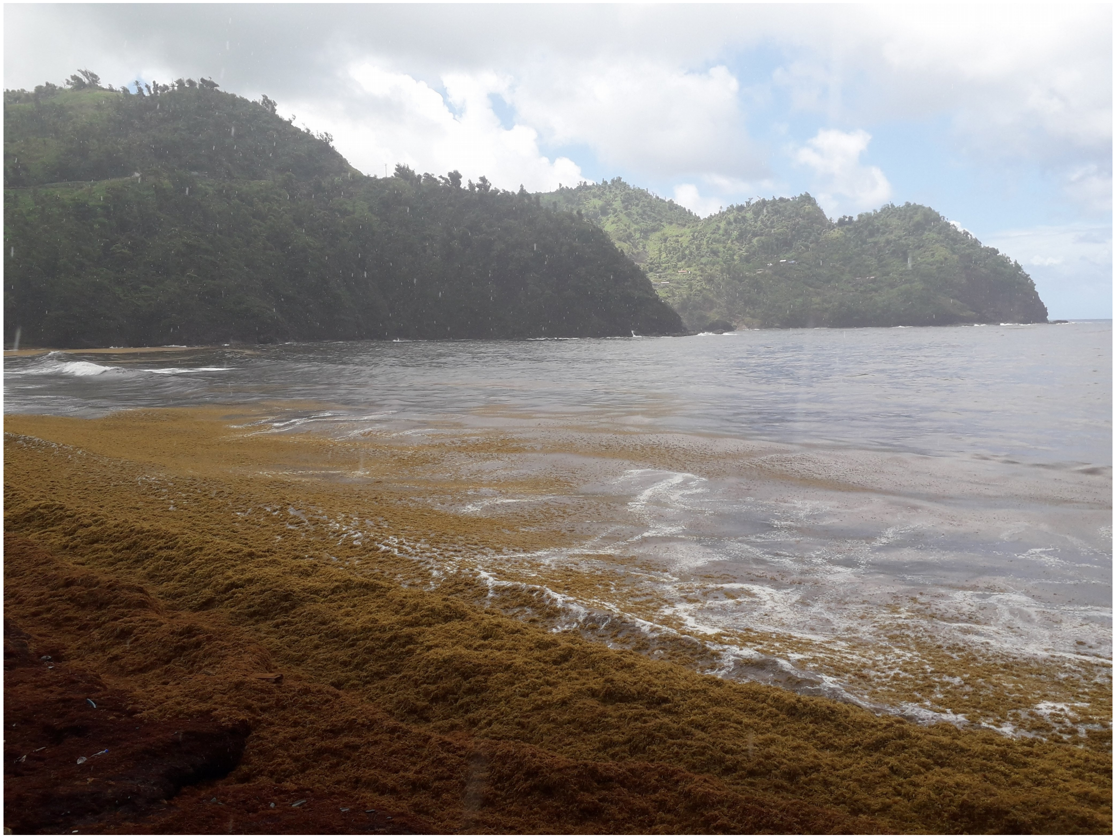

Figure S20. Pelagic *Sargassum* washed ashore at San Sauveur, Dominica. July, 2019
